# Nutritional Assessment in Older Adults: MNA® 25 years of a Screening Tool & a Reference Standard for Care and Research; What Next?

**DOI:** 10.1007/s12603-021-1601-y

**Published:** 2021-02-12

**Authors:** Yves Guigoz, B. Vellas

**Affiliations:** 1 Gerontopole, Toulouse University Hospital, INSERM UMR 1027, University of Toulouse, Toulouse, France; 2 Chemin du Raidillon, CH-1066, Epalinges, Switzerland

**Keywords:** Nutrition, MNA, aging

## Abstract

A tool to assess nutritional status in older persons was really needed. It took 5 years to design the MNA® (Mini Nutrition Assessment) tool, complete the first validations studies both in Europe and in the U.S. and to publish it. After the full MNA®, the MNA® short form and the self-MNA® have been validated. As well as Chinese and other national MNA® forms. Now more than 2000 clinical research have used the MNA® all over the world from community care to hospital. At least 22 Expert groups included the MNA® in new clinical practice guidelines, national or international registries. The MNA® is presently included in almost all geriatric and nutrition textbook and part of the teaching program for medicine and other health care professional worldwide. The urgent need is to target the frail older adults more likely to have weight loss and poor appetite and to prevent frailty and weight loss in the robust. We present in this paper the review of 30 years of clinical research and practice using the MNA® worldwide.

## Introduction and short history

Malnutrition in older adults is a really important problem, occurs 40% more often in the older ones; and is identified in 1 of 3 older adults in the hospital, and in 1 of 2 rehabilitation patients (1). Subjects intake is often poor from lack of appetite, most adults in the hospital eat less than 50% of the served food at each meal and the proportion of patients with malnutrition increases during hospital stay (2, 3).

A tool to assess nutritional status in older persons was really needed. It is why in 1989 at my first IAGG conference in Acapulco, I spoke about the MNA® idea with Yves Guigoz, from the Nestle International Research Center in Lausanne. I told him we must design and validate a tool for assessing nutritional status in the elderly analogous following the MMSE tool for assessing cognitive functions. Because already most physician know that a total score of 30 is the maximum for cognitive functions our total score for the MNA® must be also at 30.

## Validation of the MNA® full form, short form and self-management form (fig. 1)

It took us 5 years (1994) to convince our colleagues, to design the tool, to complete the first validations studies both in France and in the U.S. and to publish it (4, 5). We would like to acknowledge Werner Bauer, former director of the Nestlé Research Center who took the decision to fund the study, Phill Garry Ph.D., W.C Cameron Chumlea Ph.D., from the University of New Mexico Aging Process Study, Albuquerque, NM, USA and our team at the Gerontopole, including Sylvie Lauque RD. We validated the MNA® screening versus the results from 2 physicians with all the current nutritional assessment including nutritional intake, anthropometric measurement, and biological biomarkers (e.g. albumin, prealbumin, CRP, αl-acid glycoprotein, cholesterol, triglycerides, vitamins A, D, E, B1, B2, B6, and B12, folate, copper, zinc, haemoglobin, and blood cell count). We did it in two different populations in Toulouse area, France and in Albuquerque, NM, USA. Subjects were classified using principal component and discriminant analysis. Principal component analysis indicated that the MNA® can be used without clinical biochemistry. Threshold value ranges for risk of malnutrition and malnutrition were 22–24 points and 16–18 points, respectively, on a maximum of 30 points. Exact threshold values were then set by cross-tabulation of cut-off values for serum albumin without the presence of inflammation. We have been able to observe that those with an MNA® score less than 17.5 have usually protein-calorie undernutrition, those between 17 and 23 are at risk for malnutrition but have not yet protein-calorie undernutrition and those > 23 have in general an adequate nutritional status (5, 6, 7).

**Figure 1:**
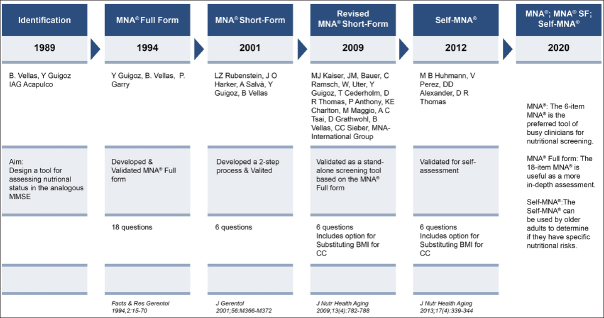
MNA®: History & Development

In 2001, With Larry Rubenstein, from U.C.L.A we developed validated the MNA® Short-form (8). The MNA® short form includes 6 items to do a first steps to screen those at risk for malnutrition. After completing the MNA® short form it is still useful if the subjects are scored at risk for malnutrition to complete the full MNA®. Carefully looking at the full MNA® items to determine where the subjects lose point can help to guide the nutrition intervention. For e.g. if we observe that a patient doesn't eat on the evening we can propose something, them if some subjects need help to eat… it takes few minutes to do the MNA® SF and it is already a very common used tool.

In 2009 a Revised MNA® short-form (9) has been developed & validated as a stand-alone screening tool; it includes option for substituting calf circumference (CC) for BMI, and takes less than 5 minutes. The MNA®-SF is available in 42 languages, https://www.MNA-elderly.com. The validity of substituting CC for BMI has been further validated (10, 11, 12).

In 2012 the Self-MNA® was developed (13) and validated in community-dwelling older adults, results shared with their family medicine (14). It takes 3–5 minutes. If we want to, improve the health of older persons self-management and participative care are very useful underlining the importance of such tool (15, 16).

### MNA® use in clinical research

As we can see in table 1, more than 2000 clinical research have used the MNA® all over the world on many topics from frailty to hip fractures, from community care to hospital (see recent publications 2018–2020 (1, 14, 15, 16, 17, 18, 19, 20, 21, 22, 23, 24, 25, 26, 27, 28, 29, 30, 31, 32, 33, 34, 35, 36, 37, 38, 39, 40, 41, 42, 43, 44, 45, 46, 47, 48, 49, 50, 51, 52, 53, 54, 55, 56, 57, 58, 59, 60, 61, 62, 63, 64, 65, 66, 67, 68, 69, 70, 71, 72, 73, 74, 75, 76, 77, 78, 79, 80, 81, 82, 83, 84, 85, 86, 87, 88, 89, 90, 91, 92, 93, 94, 95, 96, 97, 98, 99, 100, 101, 102, 103, 104, 105, 106, 107, 108, 109, 110, 111, 112, 113, 114, 115, 116, 117, 118, 119, 120, 121, 122, 123, 124, 125, 126, 127, 128, 129, 130, 131, 132, 133, 134, 135, 136, 137, 138, 139, 140, 141, 142, 143, 144, 145, 146, 147, 148, 149, 150, 151, 152, 153, 154, 155, 156, 157, 158, 159, 160, 161, 162, 163, 164, 165, 166, 167, 168, 169, 170, 171, 172, 173, 174, 175, 176, 177, 178, 179, 180, 181, 182, 183, 184, 185, 186, 187, 188, 189, 190, 191, 192, 193, 194, 195, 196, 197, 198, 199, 200, 201, 202, 203, 204, 205, 206, 207, 208, 209, 210, 211, 212, 213, 214, 215, 216, 217, 218, 219, 220, 221, 222, 223, 224, 225, 226) and MNA® and MNA®-SF tables Identifying the elderly at risk of malnutrition (Tables Table 2, Table 3, Table 4, Table 5, Table 6, Table 7, Table 8, Table 9, Table 10, Table 11, Table 12, Table 13). We observed a prevalence of malnutrition of 5% (SE 0.1) and 4.3% (SE 0.1) in the community for MNA® and MNA®-SF respectively; of 11% (SE 0.2) and 11.0% (SE 0.3) for the frail elderly (outpatients and home care) respectively. A Higher prevalence of malnutrition is observed in hospitals, 22% (SE 0.2) and 29% (SE 0.3) for MNA® and MNA®-SF; and for institutionalized elderly 18% (SE 0.3) and 22% (SE 0.4) respectively. Cognitively impaired elderly and Parkinson's disease patients have similar prevalence to the frail elderly, 14% (SE 0.4), and 6.3 (SE 0.9) for MNA®. (see Table 14: Prevalence of malnutrition and risk of malnutrition in different settings).

**Table 1:** MNA®: Publications As of October 2020, at least 2500 articles have been published using the MNA®, covering a broad range topics

**Search terms e.g. Medline**	**n Publications**
**Frailty / Sarcopenia**	
[(«Sarcopenia»[Mesh]) OR «Frailty»[Mesh] AND [«Mini Nutritional Assessment» OR MNA®-SF]	436
***Functionality***	
«Physical Fitness»[Mesh] AND [«Mini Nutritional Assessment» OR MNA®-SF]	365
**Disability**	
«Disability Evaluation»[Mesh] AND [«Mini Nutritional Assessment» OR MNA®-SF]	262
**Community**	
[«Community Health Services»[Mesh] OR «Community Medicine»[Mesh]] AND [«Mini Nutritional Assessment» OR MNA®-SF]	405
**Outpatient/Home care**	
[(«Home Care Services»[Mesh]) OR «Outpatients»[Mesh]] AND [«Mini Nutritional Assessment» OR MNA®-SF]	405
**Hospital setting**	
«Hospitals»[Mesh]AND [«Mini Nutritional Assessment» OR MNA®-SF]	387
**Nursing home / Long term care**	
[(«Nursing Homes»[Mesh]) OR «Long-Term Care»[Mesh]] AND [«Mini Nutritional Assessment» OR MNA®-SF]	483
**Cognitively impaired elderly**	
[(«Memory Disorders»[Mesh]) OR «Dementia»[Mesh]] AND [«Mini Nutritional Assessment» OR MNA®-SF]	456
**Total publications Medline**	
«Mini Nutritional Assessment» OR MNA-SF	1604

**Table 2:** MNA®-SF Clinical practice: Community-dwelling elderly — Identifying the elderly/adults at risk of malnutrition

**Setting**			**Nutritional status evaluation [% of subjects]**						**Reference**
	**n**	**Age [year]**	**Under-nourished <8**		**At risk of malnutrition 8 – 11**	**Well-nourished ≥12**	**Pub Year**	**Country**	
Free-living elderly at community pharmacists — Men	8014	75.2 ± 6.6		67		33	2009	Spain	Cuervo M. et al. (2008) Arch Gerontol Geriatr 2009;49(1):69–73 Public Health Nutr 2008;12(1):82–90
Free-living elderly at community pharmacists — Women	13993	75.2 ± 6.9							
Community dwelling patients: NutriAction Study	975	79.5± 7.2		36		64	2013	Belgium	Arvanitakis M. et al. (2013) e-SPEN Journal 2013;8: e213-e215 (Aging Clin Exp Res 2019;31:175–183
Elderly patients at inpatient ward of internal medicine and neurology	77	over 60 years old		53		47	2014	Indonesia	Prasetyo W.H. et al. (2014) Indonesian journal of nutrition and dietetics 2014;2:75 – 84
Community-dwelling older population: the VERISAÚDE study	749	75.8 ± 7,2		14		86	2016	Spain	Maseda A. e Public Health Nutr. 2016;19(12):2220–2228
Elderly living alone or on a low income in three rural regions of Korea	187	77.4 ± 5.1		78		22	2018	Korea	Jang I.-Y. et al. (2018) Clin Interv Aging 2018;13:1799–1814
Older adults aged ≥65 years, representative of the Portuguese older population: Nutrition UP 65 study	1495	74.9 ± 7.0 (65 – 100)		16		84	2018	Portugal	Mendes J. et al Sci Rep 2018;8: 4227 Sousa-Santos A.R.et al Nutr Diet 2019;76:604–612
Community-dwelling (98%) and institutionalized (2%) adults aged ≥ 65: Toledo Study on Healthy Ageing	1660	75.6 ± 6.3		26		74	2020	Spain	Rodríguez-Mañas L. et al. Clinicoecon Outcomes Res 2020;12:355–367 Clin Nutr Online 2020 Aug 4
“Survey of Health and Living Status of the Elderly in Taiwan”, population-based longitudinal cohort study: wearing fixed denture	833	≥ 65	3		18	79	2011	Taiwan	Tsai A. et al. J Nutr Health Aging 2011;15:265–270
removable-denture	1237		4		21	74			
no denture	696		6		20	74			
Home-dwelling older people, ≥65 years of age, living in five counties in southern Norway	1915	74.5 ± 6.9	2		12	87	2012	Norway	Söderhamn U. et al. Clin Interv Aging 2012;7:383–391
Elderly ≥75 years, invited for annual health assessments at a general medical practice	225	81.3 ± 4.3	0		16	83	2013	Australia	Winter J. et al. J Nutr Health Aging 2013;17:322–325
Randomly selected sample of community-dwelling older people aged ≥75	696	81 ± 4.6	1		15	84	2013	Finland	Nykänen I. et al, Eur J Public Health. 2013;23(3):405–409
Community-dwelling subjects urban environment MNA®-SF with BMI	932	71.7 ± 5.3	5		29	66	2014	Poland	Kostka J. et al. J Nutr Health Aging 2014;18:366–371 Eur J Clin Nutr 2014;68:1210–1215
MNA®-SF with Calf circumference			4		30	65			
Community-dwelling subjects rural environment MNA ®-SF with BMI	812	73.0 ± 6.6	12		37	51	2014	Poland	Kostka J. et al. J Nutr Health Aging 2014;18:366–371 Eur J Clin Nutr 2014;68:1210–1215
MNA®-SF with Calf circumference			16		39	45			
Individuals aged ≥ 75 living in the community.	640	81.3 ± 5.0	2		20	77	2014	Spain	Jürschik P. et al. Med Clin (Barc) 2014;143:191–195
Community-dwelling adults in KwaZulu-Natal	984	68.9 ± 7.4	6		43	51	2015	South Africa	Naidoo I. et al. J Health Popul Nutr 2015;33:19 Narainsamy J. et al. S Afr J Psychiatr 2015:21(1):13–18
Community elderly from the SenTo-panel, Wageningen project	345	67.1 ± 6.0	0		11	89	2015	The Netherlands	Toussaint N. et al. Chem Senses 2015;40(3):197–203
Community-dwelling older adults, random sample: Swedish National study on Aging and Care-Kungsholmen	3041	73.7 ± 10.7	2		25	74	2016	Sweden	Shakersain B. et al. Eur J Clin Nutr 2016;70:320–325
Free-living elderly in the province of Valencia selected in 12 community centres MNA®-SF BMI MNA®-SF CC	660	74.3 ± 6.6	1		27	73	2017	Spain	Montejano Lozoya R. et al PeerJ 2017;5:e3345
			2		26	72			
Community dwelling elderly	432	72 ± 10	0		69	31	2017	Taiwan	Chang S.-F. J Nurs Scholarsh 2017;49:63–72
Cross-sectional study of population-based cohort (Singapore Longitudinal Ageing Study	5697	66.6 ± 7.8	3		28	70	2017	Singapore	Wei K. et al. J Am Med Dir Assoc 2017;18(12):1019–1028
Community dwelling elderly from various settings in Tabriz (mosques, parks, organizations offering activities for older people, and advertisements)	164	74 ± 8.3	7		26	62	2018	Iran	Saghafi-Asl M. et al. Aging Clin Exp Res 2018;30:1117–1125
Home dwelling elderly individuals	407	72 ± 6	4		22	74	2018	Turkey	Acar-Tek N. &amp; KaraÇil-Ermumcu M.Ş. (2018) J Nutr Health Aging 2018;22:996–1002
Dutch community-dwelling older adults: PhysioDom HDIM project	97	78.4 ±7,2	1		20	79	2018	The Netherlands	van Doorn-van Atten M.N. Public Health Nutr 2018;22(2):363–374 British J Nur 2018;119:1185–1194
Community-dwelling persons ≥75 years of age from Alabama: University of Alabama at Birmingham (1) Study of Aging II	414	81.6 ± 4.7	12		52	37	2018	U.S.A.	Buys D.R. et al. Fam Community Health 2018;41:S33-S45
Population-based cohort study, community-dwelling adults in Singapore aged 55 years or older at baseline	2726	66.0 ± 7.7	4		31	65	2018	Singapore	Wei K. et al. JAMA Network Open 2018;1:e180650 J Nutr Health Aging 2018;22:1221–1227
Community-dwelling residents in urban and rural regions nationwide: Korean Frailty and Aging Cohort Study	1473	76.1 ± 3.9	1		14	86	2018	Republic of Korea	Kim J. et al. J Nutr Health Aging 2018;22:774–778
Patients referring to the GP offices Women	125	75.1 ± 8	11		23	66	2018	Italy	Donini L. et al. J Nutr Health Aging 2018;22:44–52
Men	101	75.3 ± 8	5		26	69.6			
Community dwelling patients: NutriAction II Study	819	82.7 ± 6.1	7		29	63	2019	Belgium	Vandewoude M.F.J. et al. Aging Clin Exp Res 2019;31:175–183 Aging Clin Exp Res 2019;31:295–298
(Sarcopenia and Physical Impairment with advancing Age)	411	73.2 ± 6.1	2		11	87	2020	Belgium	Sanchez-Rodriguez D. et al. (J Cachexia Sarcopenia Muscle 2020;11:1200–1211)
75 years or older, community-dwelling participants Village with canteen	140	83.1 ± 4.2	3		30	67	2020	China	Wang X. et al. BMC Public Health. 20204;20(1):230.
Village without canteen	144		5		31	64			
Elderly living in 10 randomly selected People's Housing Projects (PPR) flats at Kuala Lumpur	301	67.1 ± 5.5	3		30	67	2020	Singapore	Norazman C.W. et al. Nutrients 2020;12(6):1713;
Community-dwelling seniors ≥ 65 years of age from three towns in Bosnia and Herzegovina	821	74.1 ± 5.4	7		49	44	2020	Bosnia and Herzegovina	Pavlović J.R. et al. Public Health Nutr 2020;on line September 01, 2020
**Community-dwelling elderly**			**Under-nourished <8**		**At risk of malnutrition 8 – 11**	**Well-nourished ≥12**			
Total n	53617	mean	4		27	69			
		SE	0.1		0.01	0.2

**Table 3:** MNA® -SF Clinical practice: elderly — Hospial Settings — Identifying the elderly/adults at risk of malnutrition

**Setting**			**Nutritional status evaluation [% of subjects]**						
	**n**	**Age**	**Undernourished & At risk of malnutrition**			**Well-nourished**	**Pub Year**	**Country**	**Reference**
		**[year]**	**<8**		**8 – 11**	**≥12**			
Consecutive patients ≥ 60 years admitted to General surgical and Urological wards for surgery	190	69 ± 7.1		35		65	2004	Thailand	Reodecha P. et al. J Med Assoc Thai 2004;87(3):289–295
Patients admitted to a Brazilian public university hospital	516	>18 year		73		27	2010	Brazil	Raslan M. et al. Nutrition 2010;26:721–726
Patients admitted to the neurological and neurosurgical wards	196	66 ± 13		41		59	2010	The Netherlands	Hafsteinsdóttir T.B. et al. J Clin Nurs 2010;19:639–648
Hospitalised patients	101	≥ 65		97		3	2011	The Netherlands	Neelemmaat F. et al. J Clin Nurs 20:2144–52
Short-stay geriatric department in nine French hospital centers	1306	85 ± 6		74		26	2012	France	Dramé M. et al. Rev Epidemiol Sante Publique 2012;60:189–196
Patients admitted to three different internal medicine units	106	79.4		81		19	2012	Spain	Calvo I. et al. Nutr Hosp 2012;27:1619–1625
Patients undergoing Transcatheter aortic valve implantation (TAVI)	119	83.4 + 4.6		45		56	2013	Switzerland	Schoenenberger A.W. et al. Eur Heart J 2013;34:684–692
Patients ≥70 years with colorectal cancer	143	75 (70-92)		27		73	2013	The Netherlands	Aaldriks A.A. et al. Geriatr Oncol 2013;4:218–226
Elderly patients admitted for surgery	142	71.8 ± 5.4 women 72.0 ± 5.9 men		45		55	2015	China	Zhou J. et al.Nutr J 2015;14:68
Older adults admitted to rural rehabilitation facilities	57	79.1 7.3		88		12	2016	Australia	Marshall S. et al. J Acad Nutr Diet 2016;116:795–801
Ambulatory patients with chronic heart failure	130	66.3 ± 11.5		26		74	2016	Germany	Saitoh M. et al. Wien Klin Wochenschr 2016;128:497–504
Geriatric trauma patients	50	84.9 ± 5		62		38	2016	Germany	Goost H. et al. Technology and Health Care 24 (2016) 225-239
Hospitalized patients of a Department of Arthroplasty	351	67.9 (28–91)		24		76	2018	Germany	Ihle C. et al. BMC Musculoskeletal Disorders 2018;19:83
Elderly, aged ≥ 65 years, hospitalized	181	≥ 65		58		42	2018	Spain	Castro-Vega I. et al. Nutr Hosp 2018;35:351–358
Elderly patients with gastrointestinal cancer	255	76.5±4.8		48		52	2018	China	Ye X-J. et al. BMJ Open. 2018;8(4):e019750
Adult cancer patients (20-59.9 years) and elderly patients (≥ 60 years)	63	64.2 ± 12.0		68		32	2018	Brazil	Lima E.M.B. et al. Nutr Hosp 2018;35(5):1138–1144
Adult vascular surgery patients at hospital admission	322	67.6 ± 14.1		48		53	2019	Australia	Thomas J. et al. Br J Nutr 2019;122:689–697
Outpatients ≥70 years at the Emergency Department of the University Clinical Hospital of Valladolid	288	81.1 ± 6.6		64		36.1	2019	Spain	Bolado Jiménez C. et al. Nutrition 2019;66:142–1466
Patients in themedical wards of the University College Hospital (UCH), Ibadan	350	71.5 ± 8.0		5		95	2019	Nigeria	Adebusoye, L. et al. Ghana Med J 2019;53(3):210–216
Patients aged over 65 years with cardiovascular diseases	76	80		72		28	2019	Poland	Ostrowska, J. et al. J Hum Nutr Diet. 2019;32(1):119–127
Consecutive patients, admitted for the first time to the department of geriatrics	358	82 (76–86)		13		87	2020	Poland	Magnuszewski L. et al. Int J Environ Res Public Health. 2020;17(13):4768
Older patients undergoing pacemaker implantation	197	82.9 ± 4.9		46		54	2020	Switzerland	Schoenenberger A.W. et al. BMC Geriatr 2020;20:287
Patients ≥50 years admitted in medical and surgical services of six hospitals in Togo	520	61(55–70)		95		5	2020	Togo	Gbeasor-Komlanvi F.A. et al.BMC Geriatr 2020;20:507
**Setting**			**Nutritional status evaluation [% of subjects]**						
	**n**	**Age**	**Undernourished & At risk of malnutrition**			**Well-nourished**	**Pub Year**	**Country**	**Reference**
		**[year]**	**<8**		**8 – 11**	**≥12**			
Consecutive patients aged ≥65 years admitted to and discharged from a 900-bed academic hospital	5867	76.0 ± 7.0		46		54	2020	Japan	Ishida Y. et al. Geriatr Gerontol Int 2020;20:811–816
Patients undergoing transcatheter aortic valve implantation (TAVI).	288	83.5 ± 5.7		65		35	2020	Japan	Doi S. et al. ESC heart failure Online 2020 Sept 10
			**Nutritional status evaluation[% of subjects]**						
**Setting/Conditions**	**n**	**Age**	**Under-nourished**		**At risk of malnutrition**	**Well-nourished**	**Pub Year**	**Country**	**Reference**
		**[year]**	**<8**		**8-11**	**12-14**			
Patients aged 65 + y admitted to two rehabilitation hospitals	1615		40		52	8		Australia	Charlton K.E. J Nutr Health Aging 2010;4:622–628
MNA®-SF with BMI		81 ± 28					2010		
MNA®-SF with Calf circumference	1512		42		49	9			
Patients receiving geriatric inpatient care	444	85.3 ± 6.7	26		51	24	2012	Switzerland	Vischer U.M. et al. Clin Nutr 2012;31:113–117 &amp; Zekry D. et al. J Nutr Health Aging. 2012;16(3):225–230
Elderly subjects admitted to either, the surgical and the medical ICU at a tertiary care hospital	250	74.2 ± 6.8	6		20	74	2013	U.S.A.	Sheean P.M. et al. Clin Nutr 2013;32:752–757
Rehabilitation and treatment center for the elderly	49	70	61		39	0	2013	Switzerland	Reinert R. et al. Rev Med Suisse. 2013;9(406):2115–2119
Patients (<18 years old) with advanced heart failure	162	<18	31		48	22	2014	U.S.A.	Yost G. et al. Nutr Clin Pract. 2014;29(5):686–691
Acute care hospital patients on admission	1333	74 (65 – 97)	23.3		26	51	2014	Austria	Dorner T.E. et al. J Nutr Health Aging 2014;18:264–269
Inpatients diagnosed with hospital-associated deconditioning	169	79 ± 7	88		12	0	2014	Japan	Wakabayashi H. et al. J Rehabil Med 2014;46:277–282
Consecutive patients admitted to the cardiology unit of a tertiary care hospital	526	58.5 ± 12 (22 – 85)	6		64	30.4	2014	Sri Lanka	Pathirana A. K. SpringerPlus 2014;3:412
aged patients consecutively admitted to the acute geriatrics medical ward of Geriatrics and Gerontology department in Ain Shams University hospitals, Cairo	131	69.3 ± 8.2	43		47	10.7	2014	Egypt	Abd-El-Gawad W.M. et al. Clin Nutr 2014;33:1108–1116
Elderly patients hospitalized at a geriatric care hospital	141	73.5 ± 5.2	28		48	25	2015	Korea	Baek M-H. &amp; Heo Y-R. Nutr Res Pract 2015;9:637–643
Patients with Postoperative Acute Care Unit admission after general surgery, and surgery related to orthopedics and urology	150	>18 year	33		62	5	2016	Turkey	Özbilgin Ş. et al. Medicine 2016;95:e5038
Hospitalized pre-dialysis patients	33	73 ± 7	46		39	15	2016	Turkey	Buyukaydin B. et al. J Aging Res Clin Practice 2016;5(3):158–161
Patients hospitalized in the general geriatric and internal medicine units	392	84.8 ± 6.3	14		44	42	2016	Switzerland	Frangos E. et al.J Nutr Health Aging 2016;20:705–713
Older hip fracture patients, aged ≥ 65 years	472	84 (77-91)	9		42	49	2016	Finland	Nuotio M. et al. Eur J Clin Nutr 2016;70:393–398
Orthopedic and traumatology patient cohort	399	≥ 65	7		27.3	65	2017	Germany	Lambert C. et al. Nutrition 2017;37:60–67
Hospitalised trauma patients	521	54 ± 18.1	6		30	64	2017	Germany	Ihle c. et al. Z Orthop Unfall 2017;155:184–193
**Setting/Conditions**			**Nutritional status evaluation [% of subjects]**						
	**n**	**Age**	**Undernourished**		**At risk of malnutrition**	**Well-nourished**	**Pub Year**	**Country**	**Reference**
		**[year]**	**<8**		**8 – 11**	**12–14**			
Hospitalized elderly patients	248	70.0 ± 7.7	29		48	23	2017	Saudi Arabia	Alzahrani S.H. et al. BMC Geriatrics 2017;17(1):136
Hospitalized older diabetics and middle-aged patients, geriatric outpatients, and healthy elderly and young individuals	88	85.2 ± 6.2	76		24	0	2017	Spain	Sánchez-Rodríguez D. et al. Clin Nutr 2017;36:1339–1344
Patients, aged ≥ 65 who have undergone an operation for a traumatic hip fracture	71	85.4 ± 6.3	18		48	34	2017	Spain	Malafarina V.et al. Maturitas 2017;101:42–50
Elderly aged ≥ 70 years undergoing hip fracture surgery	415	84.0 ± 6.6	19		45	37	2017	Italy	Mazzola P. et al. J Am Geriatr Soc 2017;65:1222–1228
Hip fracture patients age >65 years admitted to a rehabilitation unit	218	83.5 ± 7.5	26		53	21	2017	Hong Kong China	Miu K.Y.D. and Lam P.S. Ann Rehabil Med 2017;41:1005–1012
Elderly consecutively admitted to the geriatric acute care ward	342	83.1 ± 6.8	11		45	44	2018	Germany	Pourhassan M. et al.Clinical Nutrition 2018;37:1354e1359
Elderly consecutively admitted at geriatric hospital ward	358	82.1 ± 8.2	49		49	3	2018	Germany	Pourhassan M. et al. Clin Nutr ESPEN 2018;27:100–104
Patients ≥70 years from the 20-bed Geriatric Evaluation and Management Unit (GEMU)	172	85 ± 6.4	31		49	20	2018	Australia	Dent E. et al. Nutr Diet 2018;75:11–16
Maori or Pacific ethnicity) at admission to hospital	234	83.6 ± 7.6	27		47	27	2018	New Zealand	Chatindiara I. et al. BMC Geriatr. 2018;18(1):78
Elderly > 85 years admitted to one of two hospital wards in Auckland	88	90.0 ± 3.7	28		43	28	2018	New Zealand	Popman A. et al.Nutr Diet 2018;75:52–58
Patients with a proximal femoral fracture	437	79 ± 12.8	13		35	52	2018	The Netherlands	van der Sijp M.P.L. et al. Injury 2018;49:2239–2243
Elderly patients of both sexes, diagnosed with cancer	200	72.5 ± 5.3	16		41	43	2028	Brazil	Lopes J.R. et al. Clin Nutr 2018;37(Suppl 1):S201
Hospitalized elderly patients	331	78.4 ± 9.7	14		40	46	2018	Brazil	El Kik R.M. et al. Nutr Hosp 2018;35(5):1059–1065
N-patients ≥70 years admitted for postacute rehabilitation	95	84.7 ± 6.6	27		73	0	2018	Spain	Sánchez-Rodríguez D. et al. Arch Gerontol Geriatr 2018;76:210–214
Consecutive patients admitted to convalescent rehabilitation wards									
with sarcopenia	343	80 ± 9	67		31	2	2018	Japan	Yoshimura Y. et al. Clin Nutr 2018;37:2022–2028
without sarcopenia	294	68 ± 11	11		64	25			
Patients newly admitted to a post-rehabilitation hospital	1056	70 ± 11	38		48	15	2019	Japan	Shiraishi A. et al. Geriatr Gerontol Int 2019;19:189–196
Patients with femoral neck, trochanteric, sub-trochanteric and basicervical hip fractures	205	83.5 ± 7.0	22		50	27	2019	Japan	Inoue T. et al. Clin Nutr 2019;38:220–226
Older individuals, who were consecutively admitted to a geriatric acute care ward	200	81.4 ± 6.6	31		60	9	2019	Germany	Sieske L. et al. Nutrients 2019;11:1986
Hospital admission, diagnosis of orthopedic surgery or stroke	415	81 ± 7.7	9		43	48	2019	Italy Spain	Lelli D. et al. J Am Coll Nutr. 2019 Jul;38(5):441–446
Older patients who were consecutively hospitalized to a geriatric acute care ward	233	82.1 ± 7.1	39		47	14	2019	Germany	Pourhassan M. et al. J Geriatr Psychiatry Neurol 2019;32(2):90–96
Patients ≥75 were screened in the Emergency department	50	83 (75-94)	22		78	0	2019	Ireland	Brady D. et al. Age Ageing 2019:48:iii1–iii16
All patients with cancer, aged 75 years and older, who were referred to the geriatric oncology clinic of Poitiers University Hospital	433	82.8 ± 4.8	29		47	24	2020	France	Liuu E. et al. BMC Geriatr 2020;20:295
**Setting/Conditions**			**Nutritional status evaluation [% of subjects]**						
	**n**	**Age**	**Undernourished**		**At risk of malnutrition**	**Well-nourished**	**Pub Year**	**Country**	**Reference**
		**[year]**	**<8**		**8 – 11**	**12–14**			
Patients hospitalized in an acute care hospital ≥70 years	300	79.4 ± 6.4	23		33	45	2020	Japan	Matsumoto Y. et al. Clin Nutr online 31 January 2020
Patients hospitalized in an acute care hospital <70 years	187	54 ± 13.8	11		36	63			
Elderly inpatients at a tertiary care public teaching hospitl	235	68 ± 0.4	46		50	3.4	2020	India	Rashid I. et al. Clin Epidemiol Glob Health 2020;8:91–95
Older non-cardiac surgical patients	288	74	14		34	52	2020	China	Zhao Y. et al. BMC Geriatr 2020;20:107
Cancer patients aged ≥65, in 44 hospitals in Brazil	3061	73.4 ± 6.6	33		39	27	2020	Brazil	D'Almeida C.A. et al. J Nutr Health Aging 2020;24:166–171
Adults, aged ≥ 65 years, admitted to the emergency departments at the University Hospital of Limerick: OPTI-MEND study	353	80 ± 7.0	8		28	64	2020	Ireland	Griffin A. et al. BMC Geriatr 2020;20:455
**Hospitalized elderly**			**Undernourished <8**		**At risk of malnutrition 8 – 11**	**Well-nourished 12 – 14**			
Total n	31068	mean	29		45	27			
		SE	0.3		0.03	0.3

**Table 4:** MNA® -SF Clinical practice — Frail elderly (Outpatient/Home Care) — Identifying the elderly/adults at risk of malnutrition

			**Nutritional status evaluation[% of subjects]**					
**Setting**	**n**	**Age**	**Undernourished & At risk of malnutrition**		**Well-nourished**	**Pub Year**	**Country**	**Reference**
		**[year]**	**<8**	**8 – 11**	**≥12**			
Warden controlled sheltered housing in the Blaenau Gwent area of South Wales	100	79.3 ± 6.3	17		83	2008	U.K.	Harris D.G. et al. J Hum. Nutr Diet. 2008;21:3–9
Population of elderly individuals living in community dwellings: Study of Health and Drugs in the Elderly (SHADES)	315	85 ± 7	60		40	2011	Sweden	Ernsth Bravell M. et al. Arch Gerontol Geriatr 2011;53:40–45
General practitioners outpatients on restrictive diets	95	80.5 ± 3.9	46		53.7	2012	France	Zeanandin G. et al. Clin Nutr 2012;31:69–73
Patient of similar age and sex, not following any restrictive diet	95	81.8 ± 4.8	23		76.8			
Consecutive patients ≥70 years with severe symptomatic aortic stenosis and referred for an in-hospital evaluation for TAVI	100	83.7 ± 4.6	44		56	2012	Switzerland	Stortecky S. et al. JACC: Cardiovascular Interventions 2012;5:489–496
Elderly, aged ≥ 65 years, outpatient	65	≥ 65	9		91.2	2018	Spain	Castro-Vega I. et al. Nutr Hosp 2018;35:351–358
Older adults from the clinics of a northeast US dental school	19	71.3 ± 5.2	32		68.4	2019	U.S.A.	Zelig R. et al. JDR clinical and translational research 2019;4:217–228
Elderly aged ≥65 years from the Department of Internal Medicine, Fattouma Bourguiba University Hospital (Monastir) and from a nursing home (Sousse)	141	75 (66-85)	41		59	2020	Tunisia	Hammami S. et al. PLoS One 2020;15:e0242152
			**Nutritional status evaluation[% of subjects]**					
**Setting/Conditions**	**n**	**Age**	**Undernourished**	**At risk of malnutrition**	**Well-nourished**	**Pub Year**	**Country**	**Reference**
		**[year]**	**<8**	**8 – 11**	**12–14**			
Outpatients with systolic heart failure	50	74.3 ± 6.2	8	20	72	2013	Portugal	Sargento L. et al. J Nutr Health Aging 2013;17:300–3044
Subjects admitted in the nursing homes and elderly community-dwelling ambulatory subjects	522	77.5 ± 8	24	37	39	2013	Italy	Donini L.M. et al. J Nutr Health Aging 2013;17:332–328
Geriatric day hospital of a large community hospital	190	82 (80-86)	9	36	55	2014	Germany	Schrader E. et al. J Nutr Health Aging. 2014;18(3):257–263
Community-dwelling older adults receiving home care	309	80.9 ± 7.9	15	41	44	2014	Germany	Kiesswetter E. et al. J Am Geriatr Soc 2014;62:512–517
Outpatients admitted to the Toulouse Frailty Platform								
Non-frail	30	78.1 ± 4.6	0	3	97	2015	France	Lilamand M. et al. J Nutr Health Aging 2015:19:570–574
Pre-frail	137	81.1 ± 5.6	0	16	84			
Frail	98	83.2 ± 5.7	5	39	56.1			
Patients visiting the geriatric diagnostic day clinic or outpatient clinic	138	80.9±7.6	16	44	40	2015	The Netherlands	Toussaint N. et al. Chem Senses 2015;40(3):197–203
Patients aged ≥65 years who were admitted to our geriatric medicine outpatient clinic	236	76.4 ± 7.2	20	32	48	2015	Turkey	Sarikaya D. et al. Arch Gerontol Geriatr 2015;61:56–60
Patients aged ≥65 years of a geriatric day hospital of a large community hospital in Nuremberg	190	80 (75-84)	9	36	55	2016	Germany	Schrader E.et al. J Nutr Health Aging 2016;20:918–926
Elderly, aged ≥65 years, being treated in community healthcare services in the Community of Madrid: DREAM + 65 Study	1103	79.5 ± 8.4	10	25	66	2016	Spain	Cuerda C. et al. Nutr Hosp 2016;33:263–269
Elderly aged ≥ 60 years who were living at home and using an in-home long-term care support center	227	≥ 60	13	54	33	2016	Japan	Okabe Y. et al. J Nutr Health Aging 2016;20:697–704
Veterans, homebound population of older: Veterans Affairs (VA) Home Based Primary Care (HBPC) program	2252	≥ 65	15	40	44	2017	U.S.A.	Win A.Z. et al. J Nutr Health Aging 2017;21:610–613
Community-dwelling spousal caregivers of older patients	79	79.4 ± 5.3	6	35	59	2018	Belgium	Potier L. et al. J Frailty Aging 2018;7:170–175 BMC Geriatr 2018;18:291
Hospital-based integrated CM Program for older persons at high risk for hospital readmission	791	79.9 ± 11	8	36.6	55	2018	Spain	Forcano Sanjuan S. et al. Eur Geriatr Med 2018;9:691–696
Older adults undergoing cancer care, who lived in the community and were ambulatory	202	≥ 65	33	31	36	2018	U.S.A.	Zhang X. et al. J Geriatr Oncol 2018;9:81–83
Elderly patients who presented consecutively at the Chief Tony Anenih Geriatric Centre	624	69.1 ± 7.2	2	33	65.1	2018	Nigeria	Adebusoye L.A. et al. Niger J Clin Pract 2018;21:443–450
Homecare recipients	267	82.9 ± 7.9	8	33	59	2018	Switzerland	Busnel C. et al. Rech Soins Infirm 2018;132:54–63
Elderly (60 years and above) in selected old people's homes, Lagos State	56	≥ 60	36	52	12.5	2019	Nigeria	Okoye C. et al. Niegerian J Nutr Sci 2019;40:91–98
			**Nutritional status evaluation[% of subjects]**					
**Setting/Conditions**	**n**	**Age**	**Undernourished**	**At risk of malnutrition**	**Well-nourished**	**Pub Year**	**Country**	**Reference**
		**[year]**	**<8**	**8 – 11**	**12–14**			
Persons ≥ 65 years receiving home care and/or living in a service flat or sheltered accommodation	97	78.4 ± 7.2	1	20	79.2	2019	The Netherlands	van Doorn-van Atten M.N. et al. Public Health Nutr 2019;22:363–374
Dialysis patients of the hemodialysis units	216	67±15	11	59	30.1	2020	Belgium	Holvoet E. et al. PLoS ONE 2020;15(3):e0229722
Outpatients attending a HF clinic at a university hospital	555	69 ± 11.5	3	16	81.4	2020	Spain	Joaquín C. et al. Clin Nutr Online 2020;39:3395–3401
disabled residents, aged ≥60 years, from a community of the Putuo District, Shanghai	572	66.2 ± 4.2	1	14	84.8	2020	China	Fang Q. et al. Front Med (Lausanne) 2020;7:552415
**Frail elderly Outpatient/Home Care**			**Under-nourished**	**At risk of malnutrition**	**Well-nourished**			
			**< 8**	**8 – 11**	**12 – 14**			
Total	9299	Mean	11	34	55			
		SE	0.3	0.1	0.

**Table 5:** MNA® -SF Clinical practice — Institution — Identifying the elderly/adults at risk of malnutrition

			**Nutritional status evaluation [% of subjects]**						
**Setting**	**n**	**Age**	**Undernourished & At risk of malnutrition**			**Well-nourished**	**Pub Year**	**Country**	**Reference**
		**[year]**	**<8**		**8 – 11**	**≥12**			
Elderly people living in sheltered housing	100	79.3 ± 6.3		12		88	2008	U.K.	Harris D.G. et al. J Hum. Nutr Diet 2008;21:3–9
Institutions South-West: Nursing-home residents	517	84.6 ± 9.0		55		45	2009	France	Bourdel-Marchasson I. et al. Nutrition 2009;25:155–164
Nstitutions South-West: Long-term care home residents	84	81.8 ± 10.4		90		10			
Nursing home residents: nutriaction Study	4359	84.0 ± 7.8		63		37	2013	Belgium	Arvanitakis M. et al. e-SPEN Journal 2013;8: e213-e215 (Aging Clin Exp Res 2019;31:175–183(page180))
Elderly, aged ≥ 65 years, institutionalized	375	84.2 ± 7.5	68			32	2018	Spain	Castro-Vega I. et al. Nutr Hosp 2018;35:351–358
Older men living in the veterans retirement community, Gangshan Veterans Home	354	85.4 ± 5.6	53			47	2019	Taiwan	Hsu Y.-H. et al. J Nutr Health Aging 2019;23:876–82
			**Nutritional status evaluation[% of subjects]**						
**Setting/Conditions**	**n**	**Age**	**Undernourished**		**At risk of malnutrition**	**Well-nourished**	**Pub Year**	**Country**	**Reference**
		**[year]**	**<8**		**8 – 11**	**12 – 14**			
Patients in 11 Dutch rehabilitation centres	366	55.0 <65 (n=269) ≥65 (n=97)	18		57	25	2012	The Netherlands	Hertroijs D. et al. J Rehabil Med 2012;44:696–701
Elderly from seven different residential care facilities	534	79.5 ± 7.2	16		54	29	2013	Turkey	Ulger Z. et al. J Nutr Health Aging 2013;17(4):305–309
			**Nutritional status evaluation [% of subjects]**						
**Setting/Conditions**	**n**	**Age**	**Undernourished**		**At risk of malnutrition**	**Well-nourished**	**Pub Year**	**Country**	**Reference**
		**[year]**	**<8**		**8 – 11**	**12–14**			
Institutional environment (nursing homes)									
MNA® -SF with BMI	859	79.0 ± 7.9	17		42	41	2014	Poland	Kostka J. et al. J Nutr Health Aging 2014;18:366–371 Eur J Clin Nutr 2014;68:1210–1215
MNA® -SF with Calf circumference			17		45	39			
Eight nursing homes	200	85.5 ± 7.8	17		70	14	2015	Japan	Takeuchi K. et al. PLoS One 2015;10:e0141737
Residential homes for the elderly in Lattakia	103	70.9 ± 6.4	18		42	41	2015	Syria	Hallaj F.A. East Mediterr Health J. 2015;21(10):753–761
Elderly living in 13 French nursing homes: INCUR study	773	86.2 ± 7.5	16		59	26	2015	France	Lilamand M. et al. J Nutr Health Aging 2015;19:383–388
Nursing home residents									
Women	164	82.3 ± 9	29		56	15	2016	Italy	Donini L.M. et al. J Am Med Dir Assoc 2018;17(10):959. e11-959.e18
Men	82	76.5 ± 11	22		63	15			
**Institutionalized women**									
MNA® -SF with BMI	53	44.9 ± 9.1	15		57	28	2018	Kuweit	Alkazemi D.U. et al. J Taibah Univ Med Sci 2018;13(3):238–246
MNA® -SF with Calf circumference			32		47	21			
older people living in a long-term care health facility	116	82.1 ± 7.7	73		27	7	2018	Japan	Nishida Y. et al. J Gen Fam Med 2018;19(1):9–14
Individuals living in nursing homes	773	75.9 ± 7.7	8		37	55	2019	Turkey	Basibüyük G.Ö. Et al. Maedica (Buchar) 2019;14:38–44
nursing home residents: NutriAction II Study	2480	86.3 ± 6.2	14		49	37	2019	Belgium	Vandewoude M.F.J. et al. Aging Clin Exp Res 2019;31:175–183 Aging Clin Exp Res 2019;31:295–298
Representative sample of permanent residents in long-term care homes for older adults	227	84.9 ± 6.7	17		55	28	2020	Spain	Rodríguez-Rejón A.I. et al. Nutr Clin Pract 2020;35:642–648
**Institutionalized elderly**			**Under-nourished**		**At risk of malnutrition**	**Well-nourished**			
			**< 8**		**8 – 11**	**12 – 14**			
Total	12391	Mean	22		50	28			
		SE	0.4		0.1	05

**Table 6:** MNA® -SF Clinical practice- Cognitively impaired elderly/elderly-adults with Parkinson's disease — Identifying the elderly/adults at risk of malnutrition

			**Nutritional status evaluation [% of subjects]**						
**Setting**	**n**	**Age**	**Undernourished &amp; At risk of malnutrition**			**Well-nourished**	**Pub Year**	**Country**	**Reference**
		**[year]**	**<8**		**8 – 11**	**≥12**			
**Cognitively impaired elderly**									
Elderly with mild, moderate and severe Alzheimer's disease, neurology outpatient clinic	43	80.6 ± 7.0		74		26	2018	Brazil	Santos T.N.B. et al. Nutr Hops 2018;35(6):1298–1304
Patients with diagnosis of Alzheimer type (n = 21) or mixed type (Alzheimer type plus vascular type, n = 4) dementia	25	88 (73;85)		74		26	2020	Austria	Stadlbauer V. et al. BMC Geriatr 2020;20:248
			**Nutritional status evaluation[% of subjects]**						
**Setting/Conditions**	**n**	**Age**	**Under-nourished**		**At risk of malnutrition**	**Well-nourished**	**Pub Year**	**Country**	**Reference**
		**[year]**	**<8**		**8-11**	**12-14**			
**Cognitively impaired elderly**									
Persons with dementia living in ordinary housing or special housing	1912	83.3 ± 6.4	20.2		54.1	25.7	2017	Sweden	Johansson L. et al. J Nutr Health Aging 2017 21:292–8
**Elderly/adults with Parkinson's disease**									
Community-dwelling adults with Parkinson's disease, aged >18 years	125	70.0 (35–92)	2		30	68	2013	Australia	Sheard J.M. et al. Et al. e-SPEN Journal 2013;8:e187-e92
Hospitalized elderly patients with Parkinson's disease	92	73.6 ± 6.7	7		39	54	2020	Germany	Gruber M.T. et al. PLoS One 2020;15:e0232764
**Cognitively impaired elderly Elderly/adults with Parkinson's disease**			**Under-nourished < 8**		**At risk of malnutrition 8 – 11**	**Well-nourished 12–14**			
Total	2172	Mean	10		41	49			
		SE	0.6		0.2	1.1

**Table 7:** MNA® Clinical practice- Community-living elderly — Identifying the elderly/adults at risk of malnutrition

			**Nutritional status evaluation[% of subjects]**					
**Setting**	**n**	**Age**	**Under-nourished**	**At risk**	**Well-nourished**	**Pub Year**	**Country**	**Reference**
		**[year]**	**<17**	**17 – 23.5**	**≥24**			
New Mexico Aging Process Study	330	77+6	1	18	81	1994	USA	Guigoz Y. et al. Facts, Research in Gerontology 1994;(Supp12):15–59 Vellas B. et al. Nutrition 1999;15:116–122 Scheirlinckx K. et al. Nestlé Nutr Workshop Ser Clin Perform Programme 1999;1:61–65
Community elderly city of Mataro	199	72 ± 5	1	10	90	1996	Spain	Salvà A et al. Rev Gerontol 1996;6:319–28 Nestle Nutr Workshop Ser.Clin Perform.Programme 1999;1:123–129
Non-Hispanic white elderly	420		0	15	85	1997	USA	Guigoz Y et al. Ther Umsch 1997;54:345–350
SENECA study	783	>75	1	44	55	1998	Europe	De Groot LCPGM et al. Eur J Clin Nutr 1998;52:877–83
SENECA study	171	>70	0	22	78	1999	Denmark	Beck AM et al. Ugeskr.Laeger 1997;159:6377–6381 Br J Nutr 1999;81:31–36
Hispanic elderly	356	>65	1	27	72	1999	USA	Pareo-Tubbeh SL et al J Am Diet Assoc 1999;99:572–582
Inner city African Americans	134	> 70	2	39	60	1999	USA	Miller DK et al. Nestle Nutr Workshop Ser.Clin Perform.Programme 1999;1:79–86 Morley JE et al. Nestle Nutr Workshop Ser.Clin Perform.Programme 1999;1:67–76
Random selection of elderly aged 75 years living at home in Warsaw	102	75	1	16	83	1999	Poland	Chartewska J et al. Nestle Nutr Workshop Ser Clin Perform Programme 1999;1:161
Elderly persons in the community, Jerusalem	463	>70	1	8	91	2000	Israel	Maaravi Y. et al Aging (Milano.) 2000;12:173–9
Elderly in rural and semi-rural regions of central Greece	502	74 ± 7	3	28	69	2001	Greece	Spartharakis GC et al. J Nutr Health Aging 2002;6:19
Free-living elders	97	76 (70-90)	0	33	64	2001	Chili	Urteaga C et al. Rev Méd Chile 2001;129:871–876
Elderly selected among 7 Spanish regions (Andalucia, Catalunya, Galicia, Madrid, Murcia, Navarra &amp; Valencia)	3459	73.2 ± 2	4	34	62	2001	Spain	Spanish Geriatric Oral Health Researcg Group. Int Dent J 2001;51(3):228–234 Ramon J.M. et al. Med Clin (Barc) 2001;117:766–770
Older Hispanics living indepedently	51	70 (52 – 92)	2	33	65	2002	USA	Kicklighter J.R. &amp; Duchon D. J Appl Gerontol 2002;21(1), 119-133
Home living retired elderly, Tallinn	51	51–97	0	26	74	2002	Estonia	Saava M. &amp; Kisper-Hint I.-R. J Nutr Health Aging 2002;6:93–95
Free-living elderly participating in congregate meal-site programs	69	50-90	3	32	65	2004	USA	Davidson J. &amp; al. J Nutr Elder 2004;(24)1: 53-67
Older persons living in the community	42	70.9 + 6.7	0	31	69	2004	Brazil	Delacorte R.R. et al J Nutr Health Aging 2004;8:531–534
Retires resident from a community, Shanghai	115	68 ± 9 (50 – 89)	2	19	79	2004	China	Fei X.F. et al. Chinese Journal of Clinical Rehabilitation 2004;8(21):4364–4365
Representative randomly slected elderly >53 yrs		>53				2004	Taiwan	Tsai A.C. et al. Public Health Nutr 7(1):69–76, 2004 Asia Pac J Clin Nutr 2007;16 (4):656–662 J Nutr Health Aging 2008;12(4):239–243
	910	50–60	1	8	91			
	1180	60 – 70	2	12	86			
	1820	70 – 80	4	15	81			
	530	>80	5	24	71			
Healthy free-living elderly (MNA® -SF)	150	55-85	0	0	100	2004	UK	Nayak USL &amp; Queiroga JM Gerontechnology 2004;3(2):77–88
Elderly volunters living in Ankara	1564	70 + 8	8	76	16	2005	Turkey	Kucukerdonmez O. et al. Saudi Med J 2005;26:1611–1616
«middle class» non-instutitionalised individuals aged 70-75	128	70-75	0	17	83	2005	Sweden	Eriksson B.G. et al. J Nutr Health Aging 2005;9:212–220
Community-dwellin elders	240	81.7 + 8.7 (61-93)	5	39	56	2005	USA	Chen C.C-H. et al. Adv Nursing Sci 2005;28:376–389
Active healthy elderly women	82	≥65	0	0	100	2005	France	Rolland Y. et al. J Nutr Health Aging 2005;9:397–402
Elderly free-living women	351	73 + 2.3	0	7	92	2006	Sweden	Salminen H. et al. Eur J Clin Nutr 2006;60:486–493
Independently living elderly at home	172	71.8 ± 8.4	6	19	80	2006	Austria	Hackl J.M. et al. Journal fur Ernährungsmedizin 2006;8(1):13–20
Rural elderly people	457	69 ± 8	26	62	13	2006	Bangladesh	Kabir Z.N. et al. Public Health Nutr 2006;9(8):968–974
Free living (85%) and Institutionalized (15%) elderly	238	71.5 ± 8.0	5	50	44	2007	South Africa	Charlton K.E: et al. Nutrition 2007;23(7–8):533–542
Elderly people living in one district of a city in southern Brazil	267	66.5 ± 4.2	2	20	78	2007	Brazil	Cabrera M.A. et al. J Am Med Dir 2007;8:582–584
Healthy free-living elderly	170	81.2 ± 7.4	1	36	63	2009	Italy	Buffa R. et al. Nutrition 2009;25(1):(3-5
Free living elderly, recruited using a cluster-stratified sampling method from the people of Razavi-Khorasan province	1957	70 ± 7.8	12	45	43	2008	Iran	Aliabadi M. et al. Asia Pac J Clin Nutr 2008;17(2):285–289
Free-living elderly at community pharmacists — Men	8014	75.2 ± 6.6	3	21	76	2008	Spain	Cuervo M. et al. Arch Gerontol Geriatr 2009;49(1):69–73
Free-living elderly at community pharmacists — Women	13993	75.2 ± 6.9	5	28	67	2009		Public Health Nutr 2008;12(1):82–90
Random sample of free-living elderly	471	>60 (60 – 92)	1	19	79	2008	Brazil	De Marchi R.J. et al. Nutrition 2008;24:546–553
Elderly living on the island of Sardinia	111	75.0± 7.2	1	29	70	2008	Italy	Mandas A. et al. Mediterr J Nutr Metab 2008;1:99–107
Community older adults in Wuhan	162	74.1 ± 7.95	8	36	56	2009	China	Han Y. et al. Public Health Nutr 2009;12(8):1189–1196
Representative sample of Spaniards over 65 years old	2860	73.7 ± 6.8	4	32	65	2008	Spain	Gil-Montoya J.A. et al. J Public Health Dent 2008;68(2):88–93
Elderly people from a senior college in Tokyo	130	72.2 ± 4.3	0	13	87	2008	Japan	Iizaka S. et al. Geriatr Gerontol Int 2008;8(1):24–31
2001–2006 in home-living older people, southern Sweden	579	75(2001) 80(2002)	0	15	86	2009	Sweden	Johansson et al. J Clin Nursing 2009;18(9):1354–1364
Representative sample of older people in Ourense	728	80.7 ± 7.4	13	58	30	2009	Spain	De la Montana Miguelez J.et al. Arch Latinoam Nutr 2009;59(4):390–395 J Nutr Health Aging. 2011 Mar;15(3):187–91
Polish older persons aged 65+, living in five selected regions of Poland,	420	65+	1	22	77	2010	Poland	Niedźwiedzka E. et al. Adv Med Sci 2010;55(2):172–8
Elderly people aged 60 years or older living in 19 sub-districts of the Mueang district, Phitsanuloke province	612	68.8 ± 5.9	8	67,2	25	2011	Thailand	Samnieng P. et al. J Nutr Gerontol Geriatr 2011;30:291–304
Community-dwelling elderly	83	73.7 ± 6.5	0	6	94	2012	Ireland	Claesson M.J. et al. Nature 2012:488:178–84
Community dwelling (94%), hospitalized, and nursing home residents.	175	77.8 ± 6.5	2	25	73	2012	France	Rolland Y. et al. J Am Med Dir Assoc. 2012;13(1):31–34
Community-dwelling elderly fully dentates	50	70.1 ± 6.1	0	0	100	2012	France	Cousson P.Y. et al. Gerodontology. 2012 Jun;29(2):e685-e692.
Community-dwelling elderly complete denture wearer	47	70.1 ± 8.1	2	19	79			
Quasi-probabilistic sample of people ≥ 60 years old: “Thousand's Study”								
Women	570	71 ± 7.7	9	51	41	2012	Mexico	Rodriguez-Tadeo A. et al. J Nutr Health Aging 2012;16:426–431
Men	190	73.7 ± 7.9	6	51	43			
Community-dwelling volunteer older adults	206	83 (75-96)	0	15	85	2013	Germany	Bollwein J. et al. J Nutr Health Aging 2013;17:351–356
Geriatric people aged 60 years and above in four Union Councils of Sargodha city	380	≥60	6	42	52	2013	Pakistan	Ghani A. et al. Int J med Appl health. 2013;1(1):22–28
Volunteer community-dwelling elderly people aged 65–88 year, Luozi city, rural area located in Bas-Congo province, DRC	370	69.9 ± 5.6	28	58	14	2013	Democratic Republic of Congo	Andre M.B. et al. Geriatr Gerontol Int 2013;13:35–42
Random sample of elderly in rural El-Burgaia village	350	67.7± 6.6	9	30	62	2013	Egypt	Mahfouz E, et al. J Aging Res Clin Pract 2:300–202
Elderly community-dwelling ambulatory subjects								
women	277	75.6 ± 7	15	39	47	2013	Italy	Donini L.M. et al. J Nutr Health Aging 2013;17:332–328
Men	125	76.1 ± 6	2	36	62			
Community-dwelling subjects urban environment	932	71.7 ± 5.3	2	30	68	2014	Poland	Kostka J. et al. J Nutr Health Aging 2014;18:366–371 Eur J Clin Nutr 2014;68:1210–1215
Rural environment	812	73.0 ± 6.6	8	41	52			
Elderly aged ≥ 60, representative of the urban population: SABE Study								
Men	437	69.6 ± 0.6	2	26	73	2014	Brazil	da Silva Alexandre T. et al. J Nutr Health Aging 2014;18:284–290
Women	712		1	30	69			
Healthy controls selected from the medical staff and the patients' relatives	145	59.6 ± 8.8	6	29	65	2014	Iran	Fereshtehnejad S.-M. et al. J Parkinsons Dis 2014;4:473–81
Independent elderly attending two primary care clinics in Beirut, Lebanon	202	72 ± 6	9	34	58	2014	Lebanon	El Osta N. et al. Clin Nutr 2014;33:316–21
Community-dwelling elderly, recipients of the Public Assistance (PA) scheme for socio-economically disadvantaged Singaporeans	399	76.0 ± 7.8	3	50	47	2014	Singapore	Koo Y.X. et al. Public Health Nutr 2014;17(12):2834–2843
Free living and institutionalized subjects from Kochi, Kerala	296	>60	7	40	53	2014	India	Shilpa J. &amp; Kumari K.S. Int J of Adv Res 2014;2:214–221
Individuals aged ≥ 75 years living in the community.	640	81.3 ± 5.0	2	20	78	2014	Spain	Jürschik P. et al. Med Clin (Barc) 2014;143:191–195
Community dwellers ≥ 65 years, randomly recruited: Three-City (3C) study	6040	73.5 ± 5.2	0	12	88	2015	France	Torres M.J. et al. Osteoporos Int 2015;26:2157–2164
Elderly, aged ≥ 60, randomly selected from the chosen cities of 5 provinces representing the country (geography, climate, ethnicity, and culture)	1338	69.1 ± 7.3	6	41	53	2015	Iran	Tanjani P.T. et al. Arch Gerontol Geriatr 2015;60:281–287
Elderly >60 years of age residing in the villages for longer than 6 months	360	>60	15	55	30	2015	India	Agarwalla R. et al. J Family Community Med. 2015;22(1):39–43
Independent-living older adults with mild to moderate limitations in physical function: PROVIDE study	380	77.7 ± 9.8	1	9	91	2015	Belgium Germany Ireland Italy Sweden U. K.	Bauer J.M. et al. J Am Med Dir Assoc 2015;16:740–747
Older adults ≥ 65 years, living independently, recruited in the Taipei, Taiwan region								
Nonfrail (n = 91)	152	80.9 ± 7.7	0	28	72	2016	Taiwan	Chang S.-F. et al. J Clin Nurs 2016;25:424–433
Prefrail (n = 61)			8	46	46			
Community-dwelling elderly in cities in Latin America (SABE-study)						2016		Lera L. et al. J Nutr Health Aging 2016;20:797–805
Sao paulo (brazil) women	1449	73.3 ± 8.4	3	35	62		Brazil	
Men	569		3	32	66			
Santiago (chile) women	1160	71.5 ± 8.0	1	15	84		Chile	
Men	403		1	9	90			
Havana (cuba) women	1220	72.0 ± 8.8	8	42	50		Cuba	
Men	413		5	33	61			
Mexico df (mexico) women	817	69.7 ± 7.6	3	34	63		Mexico	
Men	310		2	34	64			
Montevideo (uruguay) women	880	71.2 ± 7.3	3	33	65			
Men	294		2	27	71		Uruguay	
Elderly population living in urban community of Hawassa city	548	69.0 ± 7.0	28	62	9	2016	Ethiopia	Hailemariam H. et al. BMC Nutrition 2016;2:11
Community-dwelling elderly residing in Okharpauwa Village Development Committee	242	69.8 ±7.4	24	65	11	2017	Nepal	Ghimire S. et al. PLoS One 2017;12:e0172052
Non-institutionalized inhabitants aged ≥75 years	102	81.3 ± 4.6	2	21	77	2017	Spain	Hernández-Galiot A. &amp; Goñi I. Nutrition 2017;35:81–86
Free-living elderly in the province of Valencia selected in 12 community centres	660	74.3 ± 6.6	0	23	77	2017	Spain	Montejano Lozoya R. et al Peer J 2017;5:e3345
Community-dwelling Indonesians from both rural and urban areas of Yogyakarta								
Rural area	203	74 ± 7	3	73	24	2017	Indonesia	Arjuna T. et al. Nutrients 2017;9:1240
Urban area	324		6	44	50			
Persons aged ≥60 years permanent resident in urban and rural areas of Dharwad district in Karnataka								
Rural area	102	67 ± 7.1	28	40	31	2017	India	Ananthesh B.G. et al. Int J Community Med Public Health 2017;4:51–58
Urban area	102		9	37	54			
Older adults in a community in Pathanamthitta district of Kerala	129	≥ 60	12	47	42	2017	India	Abraham J. et al. Int J Res Med Sci 2017;6:210–215
Households with members aged 65 to74 y	287	69.3 ± 3.5	3	45	52	2018	Brazil	Stoffel L.M.B. et al. Nutriton 2018;55-56;104-110
Community-dwelling individuals ≥60 years living in the Brazilian state of Minas Gerais	2868	≥60	2	28	71	2018	Brazil	Damião R. et al. J Nutr Health Aging 2018;22(1):111–116
Community dwelling elderly from various settings in Tabriz (mosques, parks, organizations offering activities for older people, and advertisements)	164	74 ± 8.3	1	27	72	2018	Iran	Saghafi-Asl M. et al. Aging Clin Exp Res 2018;30:1117–1125
Elderly of Jahandidegan Council	180	65.4 ± 7.5	1	13	86	2018	Iran	Abdollahzade S.M. et al. Int J Nutr Sci 2018;3(2):86–91
Rural primary health care centre	169	75.1 ± 7.0	5	43	52	2018	Lithuania	Spirgiené L. et al. Int J Nurs Pract. 2018;24:e12688
Community-dwelling persons 75+ years of age from Alabama	414	≥ 75	3	41	56	2018	U.S.A.	Buys D.R. et al. Fam Community Health 2018;41:S33-S45
Elderly population in four villages of rural Puducherry	279	69.4 ± 8.1	18	59	23	2018	India	Krishnamoorthy Y. et al. J Family Med Prim Care 2018;7(6):1429–1433
Patients referring to the GP offices								
Women	125	75.1 ± 8	8	33	59	2018	Italy	Donini L. et al. J Nutr Health Aging 2018;22:44–52
Men	101	75.3 ± 8	4	31	65			
Home dwelling elderly individuals	407	72 ± 6	6	40	54	2018	Turkey	Acar-Tek N. &amp; KaraÇil-Ermumcu M.Ş. J Nutr Health Aging 2018;22:996–1002
Elderly randomly enrolled from seven different Greek cities	2092	75.0 ± 8.4	11	35	54	2018	Greece	Mantzorou M, et al. Nutr Neurosci 2018;23(3):201–209
Cross-sectional analysis, Portuguese older adults age ≥65 years: Nutrition UP 65 study	1493	74.0 (65 – 100)	1	15	84	2018	Portugal	Sousa-Santos A.R. et al. Food Nutr Bull 2018;39(3):487–492 Nutr Diet 2019;76:604–612
Elderly people in the city of Voronezh and the Region	160	69.4 ± 7.2	2	47	52	2018	Russia	Skrebneva A.V. et al. Voprosy pitaniia [Problems of Nutrition] 2018; 87 (6):42–47
Community-dwelling persons ≥75 years of age from Alabama: University of Alabama at Birmingham Study of Aging II	414	81.6 ± 4.7	3	41	56	2018	U.S.A.	Buys D.R. et al. Fam Community Health 2018;41:S33-S45
Home-living old-age population in Hong Kong	613	78.5 ± 7.4	1	28	71	2019	China	Wong M.M.H. et al. BMC Geriatrics 2019;19(1):138
Comunity very old residents of Veranópolis, Rio Grande do Sul, Brazil	153	86 ± 4	0	7	94	2019	Brazil	Senger J. et al. J Nutr Health Aging. 2019;23(10):923–929
Older adults was recruited from community recreational centres for the elderly	211	72.4 +/- 8.5	5	48	46,4	2019	Greece	Grammatikopoulou M.G. Maturitas 2019;119:8–13
Community dwelling elderly, aged ≥6 years	326	68.8 ± 7.2	3	25	72	2020	Iran	Bakhtiari A. et al. BMC Geriatr 2020;20:278
Older People Living in the Community	100	74.9 ± 8.50	2	25	73	2020	Poland	Piglowska M. et al. Nutrients 202;12(7): 2042
Nationally representative sample of communitydwelling older adults (≥ 65), PEN-3S-Study	1120	76 ± 8	0.5	16	83	2020	Portugal	Madeira T. et la. Acta Med Port. 2020;33(7-8):475–482 Nutrition 2020;73:110660
**Community-dwelling elderly**			**Under-nourished**	**At risk of malnutrition**	**Well-nourished**			
			**<17**	**17 – 23.5**	**≥24**			
Total	12391	Mean	5	31	64			
		SE	0.1	0.2	0.2

**Table 8:** MNA® Clinical practice- Hospital Settings — Identifying the elderly/adults at risk of malnutrition

			**Nutritional status evaluation[% of subjects]**					
**Setting**	**n**	**Age**	**Under-nourished**	**At risk**	**Well-nourished**	**Pub Year**	**Country**	**Reference**
		**[year]**	**<17**	**17 – 23.5**	**≥24**			
Acute care Elderly patients admitted for acute medical pathology in two geriatric units of a regional hospital	39	79 + 9	17	58	24	1997	Belgium	Gazotti C et al. J Nutr Health Aging 1997;1:23–27
Geriatric medicine Assessment on admission to hospital	166	>70	15	33	52	1999	Switzerland	Quadri P et al.Nestle Nutr Workshop Ser Clin Perform Programme 1999;1:141–147
Acute care elderly patients	151	83.8 (70-99)	26	52	22	1999	Belgium	Joosten E et al.Aging (Milano) 1999;111:390–394
Acute care General surgery and neurosciences	152	>65	15	44	41	1999	Canada	Azad N et al. CMAJ 1999;161:511–515
Acute care Elderly patients, not institutionalized, scheduled for elective surgery	419	72 (60 – 98)	7	25	68	1999	France	Cohendy R et al. Nestle Nutr Workshop Ser.Clin Perform.Programme 1999;1:117–121
Acute care	299	83± 8	24	45	31	1999	France	
Sub-acute care	196		32	55	13			Compan B et al. J Nutr Health Aging 1999;3:146–151
Long-term care	423		25	50	25			
Geriatric Medicine Elderly patients admitted to the regional university hospital	175	80+8	22	49	30	2000	Belgium	Gazzotti c et al. J Nutr Health Aging 2000;4:176–81
Acute care Orthopaedic ward patients admitted for emergency surgery	49	60–103	16	47	37	2000	U.K.	Murphy MC et al. Eur J Clin Nutr 2000;54:555–562
Internal medicine	101	79.7 ± 6.3 (70-93)	8	46	47	2000	France	Clement A et al. Presse Med 2000;29:1207–1213
Geriatric medicine Assessment on admission to hospital	1145	84.2	19	60	21	2001	Switzerland	van Nes MC et al. Age and Ageing 2001;30:221–226
Hospital, general medicine	408	63 (>60)	19	43	38	2001	France	Gin H. et al. Cah Nutr Diét 2001;36:185–188
Hospital, surgery	113		21	44	35			
Hospital, geriatry	75		53	41	6			
Geriatric ward of a general hospital	126		31	51	18	2002	Belgium	Pepersack T et al. J Nutr Health Aging 2002;6:306–310
Acute geriatric inpatient ward.	83	83+7	26	56	18	2002	Sweden	Persson M. et al. J Am Geriatr Soc 50:1996–2002, 2002
Sub-acute care	837	76 + 13	29	63	9	2002	USA	Thomas D.R. &amp; al. Am J Clin Nutr 2002;75:308–313
Geriatric hospital admissions	486	81 ± 8	74	23	3	2002	Italy	Donini L.M. &amp; al. J Nutr Health Aging 2002;6(2):141–146
Geriatric hospital	167	82 ± 8	2	30	68	2003	Italy	Donini L.M. &amp; al. Journal of nutrition 2003;7(5):282–292
Demented patients admitted to an Alzheimer section	174	80.24 ± 8.09	36	48	17	2003	Italy	Magri F. &amp; al. Aging Clin Exp Res2003; 15(2):148–153
Patients with various forms of advanced cancer about to start palliative chemotherapy	71	> 65	13	63	24	2003	Australia	Slaviero KA. &amp; al. Nutrition and Cancer 2003;(46)2:148–157
Patients admitted to 5 regional hospital	43	78.6 (68 – 94)	21	29	50	2003	Australia	Barone L. et al. J Nutr Health Aging 2003;7(1):13–17
Patients over 60-year admitted in hematology department	123	74 (60 – 97)	13	36	51	2003	France	Bauduer F. et al. J Nutr Health Aging 2003;7(3):179–182
Inpatient geriatric service of an university hospital and a geriatric ward of a non-academic teaching hospital (MNA® -SF)	298	> 60	61	32	7	2004	The Netherlands	Rypkema G. &amp; al. J Nutr Health Aging 2004;8(2):122–127
Older men with prostate cancer	total = 80	> 65 (65-94)				2004	Lithuania	Toliusiene J. &amp; al. Medicina 2002;38:929–932
Group a: advanced	40		10	50	40			
Group b: benign	40		0	8	37	2004	Spain	Support Care Cancer 2004(12):716–719
Geriatric convalescence unit (intermediate care facility)	118		46	47	8			Arellano Perez M. &amp; al. Rev Mult Gerontol 2004;14(5):258–261
First visit to a geriatric clinic for surgery	204	77.5 ± 6.1	8	37	56	2004	Spain	Esteban M. et al. Rev Esp Geriatr Gerontol 2004;39(1):25–28
Patients on discharge from surrounding acute hospitals. Hampstead Rehabilitation Centre in Adelaide, a sub-acute care facility	65	>65	29	46	25	2004	Australia	Visvanathan R. &amp; al. Age Ageing 2004;33:260–265
Geriatric Oncology Program: Cancer patients	135	78 (66-92).	24	41	36	2004	France	Terret C. et al. J Clin Oncol 2004;22(14S):8167
Rehabilitation unit at the Repatriation General Hospital	133	81+6	6	47	47	2005	Australia	Neumann S.A. et al. J Hum Nutr Dietet 2005;18:129–136
Patients attending the medical oncology day centers	157	65 (32-81)	9	57	34	2005	Australia	Read J.A. et al. Nutr Cancer 2005;53:51–56
Acute care geriatric wards	80	80.2 + 7.7	33	38	30	2005	Germany	Bauer J.M. et al. Z. Gerontol Geriatr 2005;38:322–327 ClinNutr 2005;34(4):557 (Abstr P046)
Patients admitted to hospital	200	81± 7	50	38	13	2005	Spain	Gomez Ramos et al. Nutr Hosp 2005;20(4):286–292 Arch Latinoam Nutr 2005;55(1)71-76
Patients admitted to hospital	145		68	30	2	2005	Spain	Izaola O. et al. An Med Interna 2005;22(7):313–316
Elderly hospitalized for different medical and/or surgical reasons	207	74.3 ± 7.0	9	30	61	2005	Portugal	Martins C.P.A.L. et al. J Nutr Elderly 2005;25:5–21
Prospective cohort study of patients from a geriatric hospital	414	>75	49	33	17	2005	Israel	Kagansky N. et al. Am J Clin Nutr 2005;82:784–791
Elderly institutionalized in geriatric units	126	60 – 96	6	48	46	2005	Vemezuela	Rodriguez N. et al. Invet Clin 2005;46(3):219–228
Patients aged ≥ 65 at the time of admission	108	73.1 ± 5.8	22	41	37	2005	Korea	Chung S.H. &amp; Sohn C.M. Korean J Community Nutr 2005;10:645–653
Patients referred to hospital	120	80 ± 7	17	44	39	2005	China (Hong Kong)	Shum N.C. et al. Hong Kong Med J 2005;11(4):234–242
Acute care	204	73.8 ± 5.6	1	39	60	2006	Israel	Castel H. et al. J Am Coll Nutr 2006;25:128–134
Patient who underwent major elective surgery	202	55.3 ± 14.9	17	73	10	2006	Turkey	Kuzu M.A. et al. World J Surg 2006;30:378–390
Hemodialysis Patients with CRP<10mg/dl	137	41 ± 12	13	77	47	2006	Turkey	Afsar B. et al. J Renal Nutr 2006;16(3):277–282
All consecutive patients admitted into the clinic	102	62 ± 19 (16–91)	12	38	50	2006	Switzerland	Gehring N. et al. Swiss Med Wkly 2006;136:664–669
Elderly non-diabetic patients on admisson to hospital	29	86 ± 5.8	21	59	21	2006	Switzerland	Bonin-Guillaume S. et al. Diabtes Metab 2006;32:236–243
Hospitalized patients	41	63 ± 22	51	42	7	2006	Spain	Villamayor Blanco L. et al. Nutr Hosp 2006;21(2):163–172
Admission to surgery unit	133	22 – 93	7	31	62	2006	Spain	Agalés M. et al. Nutr Hosp 2006;21(Supl 1):28(Abstr)
Hospitalized patients (Surgery &amp; Internal Medicine)	400	67 ± 16 (20 – 102)	15	44	42	2006	Spain	Velasco Gimeno C. et al. Nutr Hosp 2006;21(Supl 1):21(Abstr)
Hospitalized elderly from nine hospitals	213	73.5 ± 15	24	50	26	2006	Spain	de Luis D. et al. Eur J Intern Med 2006;17(8):556–560
Geriatric hospital population	100	65 – 89	29	52	19	2006	India	Manral M. Abstr 6th Intern Conf on Dietary Assessment Methods, Copenhagen, April 27–29, 2006
Hospital older patients	114	75.22 ± 6.29	4	43	53	2007	Taiwan	Chen C.C-H. et al. J Clin Nursing 2007;16(11):2015–2026
Hospital geriatric service	197	60 (60-75: 55%; 75-85: 35%; >85: 10%)	32	49	19	2007	Cuba	Cuyac L. M. &amp; Santana P. S. Arch Latinoam Nutr 2007;57(3):255–265
Older patients admitted to the internal medicine department	589	> 65	19	36	57	2007	Israel	Feldblum I. et al. Nutr J 2007;6:37
Multimorbid geriatric patients in acute care with Pressure Ulcere	81	79.3 ± 7.4	40	58	3	2007	Germany	Hengstermann S. et al. J parenter Enteral Nutr 2007; 31:288–294
Idem, without Pressure Ulcer	403	79.7 ± 7.7	17	60	24			
Rehabilitation ward	38		4	42	54	2007	Australia	Neumann S.A. et al. Nutr Dietetics 2007;64:179–185
Hip fracture patients	157	>75	10	60	30	2007	Sweden	Olofsson B. et al. J Clin Nursing 2007;16:2027–2038
Admission to cardiac and orthopaedic services	114	75 ± 6	4.4	43	52.6	2007	Taiwan	Chen C.C-H. et al. J Clin Nursing 2007;16:2015–2026
Consecutively admitted patients	205	82 (75 – 95)	30	60	10	2007	Germany	Saeglitz C. PhD Thesis 2007, Hohen Landwirtschaflichen Falkultät der Rheinischen Friedriech-Wilhems-Universität Bonn
Consecutively admitted patients	97	71 ± 7.7	18	50	32	2007	Mexico	Gutiérrez Reyes J.G. et al. Nutr Hosp 2007;22(6):702–709
Hospitalized elderly	123	>65	24	42	35	2007	Spain	Villar Taibo R. et al. Nutr Hosp 2007;22(Supl 1):68(Abstr)
Admission to orthopedic surgery	107	62 ± 20 (16 – 95)	12	22	66	2008	Spain	Garcia Duque S. et al. Nutr Hosp 2008;23(5):493–499
Admisson to hospital	41	83 ± 5	29	71	0	2008	Spain	Trabal J. et al. Clin Nutr 2008;Suppl3(1):61(P078)
Admisson to hospital: elderly patients	531		32	51	17	2008	Portugal	Cansado P et al. Clin Nutr 2007; Suppl2(2):99(P032)
Elderly patients at a tertiary teaching hospital	100	81.9 ± 6.3	30	61	9	2008	Australia	Adams N.E. et al. Nutr Dietetics 2008;65:144–150
Multimorbid patients	808	77.1 ± 9.0	20	65	15	2008	Germany	Hengstermann S. et al. J Nutr Health Aging 2008;12(2):117–122
Mono-centre non-interventional trial hospitalised patients	102	62 ± 19	12	38	50	2006	Switzerland	Gehring N. et al. Swiss Med Wkly 2006;136(41-42):664–669
Patients hospitalized for pneumonia in the Acute Geriatric Unit of Hospital de Mataró, Barcelona	117	84.7 ± 6.5	31	53	16	2008	Spain	Cabré M. et al. Med Clin (Barc) 2008;131(5):167–170
Patients with rheumatoid arthritis at inpatient ward	59	65 (55 – 75	22	51	27	2008	Sweden	Elkan A.C. et al. Eur J Clin Nutr 2008;62:1239–1247
COPD patients admitted to an acute care hospital ward	50	75.7 ± 6.9	48	48	2	2008	Sweden	Odencrants S. et al. J Clin Nursing 2008;17:1771–1778
Hospitalized medical patients	195	>65	0	39	61	2008	Israel	German L. et al. J Nutr Health Aging 2008;12(5):313–318
180 persons, 65 males and 115 females (Rehabilitation unit)	180	79.5 (65-97)	19	67	14	2008	Italy	Amici A. et al.Arch Gerontol Geriatr 2008;46(3):327–334
Admission to medicine guard of a military hospital	113	78.3 ± 7.7 (65 – 98)	38	41	21	2008	Chile	Hirsch S. et al. Open Longevity Sci 2008;2:17–22
Unilateral lower-extremity amputees, Prosthetics clinic	58	66 (21 – 91)	2	28	71	2008	Australia	Miller M. et al. Arch Phys Med Rehabil 2008;89:2031–2033
Patients with metastatic lung cancer	163	65.4	26	46	28	2008	Greece	Gioulbasanis I. et al. J Clin Oncol 2008;26(15S Suppl):19062
Older hospitalised patients aged ≥ 65 years at hospitalization	306	71.8 (65 – 89)	9	64	28	2009	Taiwan	Chen C.C.H. et al. J Clin Nurs 2009;18:3299–3307
After 16 days hospitalisation/Discharge			37	54	9			
Elderly patients with hip fractures	32	66-95	44	53	3	2009	Sweden	Wengstrom Y. et al. J Nutr Health Aging 2009;13:632–638
Consecutively admitted patients	432	63 ± 19	10	20	70	2009	Switzerland	Venzin R.M. et al. Eur J Clin Nutr 2009;63:430–436
Elderly hospitalized in a hospital that provides care for the public and private healthcare systems	240	≥ 60	29	37	34	2009	Brazil	Oliveira M. et al. Nutr J 2009;8:54
Inpatients admitted to an acute geriatric ward	104	84 (78-89)	22	48	30	2010	Switzerland	Drescher T. et al. Eur J Clin Nutr 2010;64:887–893
Patients admitted to Internal Medicine	143	65.2 ± 16.5	10	53	37	2010	Spain	Bernabeu-Wittel M. et al. Arch Gerontol Geriatr. 2010;51(2):1851–1891
Patients admitted to the neurological and neurosurgical wards	196	66 ± 13	7	34	59	2010	The Netherlands	Hafsteinsdóttir T.B. et al. J Clin Nurs 2010;19:639–648
Elderly patients ≥ 75 from all Belgian general and teaching hospitals with elderly wards	2329	83.8 ± 5.2	33	43	24	2010	Belgium	Vanderwee K. et al.Clin Nutr 2010;29:469–476
Patients aged 65 + y admitted to two rehabilitation hospitals	2076	80.6 ± 27.7	33	52	16	2010	Australia	Charlton K.E. et al. J Nutr Health Aging 2010;4:622–628
Patients >18 years old, with newly diagnosed metastatic lung cancer, admittedto the Department of Medical Oncology, University Hospital of Heraklion	115	66 (32-86)	25	51	24	2011	Greece	Gioulbasanis I. et al. Lung Cancer 2011;74(3):516–520
Patients were randomly selected from the hospital admission register	400	67.4 ± 16.1	15	44	42	2011	Spain	Velasco C. et al. Eur J Clin Nutr. 2011;65:269–274
Patients with cancer >70years for whom chemotherapy was prescribed by their medical oncologist	202	77 ± 4.2	3	30	65	2011	The Netherlands	Aaldriks A.A. et al. Crit Rev Oncol Hematol 2011;79:205–212
Older non-diabetic and diabetic patients	164	85.2 ± 6.4	17	53	29	2012	Switzerland	Vischer U.M. et al. Clin Nutr 2012;31:113–117
Patients admitted to three different internal medicine units	106	79.4	22	55	24	2012	Spain	Calvo I. et al. Nutr Hosp 2012;27:1619–1625
Patients admitted in different medical or surgical wards	57	70.5 ± 16	14	35	51	2012	Spain	Ocón Bretón M.J. et al. Nutr Hosp 2012;27:701–706
Elderly at admission to the geriatric evaluation and management unit at The Queen Elizabeth Hospital in Adelaide	100	85.2 ± 6.1	40	44	16	2012	Australia	Dent E. et al. J Nutr Health Aging 2012;16:764–767
Older medical patients, large metropolitan public teaching hospital	134	80 ± 8	32	38	30	2013	Australia	Young A.M. et al. Nutrition 2013;29:101–106
People 65 years of age admitted to a medium-sized Swedish hospital	1767	78.1 ± 7.8	9	55	36	2013	Sweden	Söderström L. et al. Clin Nutr 2013;32:281–288
Elderly subjects admitted to either, the surgical and the medical ICU at a tertiary care hospital	250	74.2 ± 6.8	10	24	66	2013	U.S.A.	Sheean P.M. et al. Clin Nutr 2013;32:752–757
Older patients, older than 70, with cancers or lymphoma during chemotherapy	606	78.0 ± 4.9	13	52	35	2014	France	Bourdel-Marchasson I. et al. PLoS One 2014;9:e108687 PLoS One 2016;11:e0148523
Aged patients consecutivelyad-mitted to the acute geriatrics medical ward of Geriatrics and Gerontology department in Ain Shams University hospitals, Cairo	131	69.3 ± 8.2	41	53	7	2014	Egypt	Abd-El-Gawad W.M. et al. Clin Nutr 2014;33:1108–1116
Patients (>18 years old) with advanced heart failure	162	>18	25	65	10	2014	U.S.A.	Yost G. et al. Nutr Clin Pract. 2014;29(5):686–691
Consecutively admitted internal medicine patients at Sakarya Education and Research Hospital	130	76.2±7.2	37	31	32	2015	Turkey	Demir M.V. et al. Niger J Clin Pract. 2015;18(6):757–761
Elderly patients hospitalized at a geriatric care hospital	141	73.5 ± 5.2	26	40	34	2015	Korea	Baek M.-H. and Heo Y.-R. Nutr Res Pract 2015;9:637–643
Hospitalized patient of a tertiary hospital center	194	71.6 ± 21.4	23	46	31	2015	Spain	Calleja Fernández A. et al. Nutr Hosp 2015;31:2240–2246
Geriatric trauma patients	50	84.9 ± 5	28	42	30	2016	Germany	Goost H. et al. Technology and Health Care 2016;24: 225-239
Acute geriatric ward admission	120	82.5 ±8.0	27	48	25	2016	Norway	Jacobsen L. et al. BMJ Open 2016;6:e011512.
Patients with the age of 75 years or older and an indication for hip surgery	226	83 ± 5	5	27	68	2016	The Netherlands	van Wissen J. et al. J Nutr Health Aging 2016;20:964–968
Older adults admitted to rural rehabilitation facilities	57	79.1 ± 7.3	28	58	14	2016	Australia	Marshall S. et al. J Acad Nutr Diet 2016;116:785–794
Patients aged ≥70 years admitted to the Geriatric Evaluation and Management Unit following a brief acute hospital stay	100	85.2 ± 6.1	40	44	16	2017	Australia	Dent E. et al. Australas J Ageing 2017;36:E8-E13
Older patients (aged ≥ 60 years) from the acute geriatric wards of two hospitals in Chengdu	453	79.0 ± 7.8	10	41	48	2017	China	Hu X. et al. Sci Rep 2017;7:3171
Orthopedic and traumatology patient cohort	398	≥ 65	4	34	62	2017	Germany	Lambert C. et al. Nutrition 2017;37:60–67
Hospitalized patients in an internal medicine clinic: Adult	84	54 ± 7	7	33	60	2017	Turkey	Yürüzer, M. et al. Turk J Med Sci 2017;47:1362–1369
Elderly	112	77 ± 8	17	68	15			
Hospitalised trauma patients	521	54.0 ± 18.1	3	33	65	2017	Germany	Ihle c. et al. Z Orthop Unfall 2017;155:184–193
Adult patients (>18 years) with solid tumor diagnosis and a life expectancy >3 months	1952	62.7 ± 12.9	9	42	49	2017	Italy	Muscaritoli M. et al. Oncotarget 2010;8:79884–79896
Acutely ill patients admitted to a geriatric ward of a tertiary university hospital in São Paulo	1409	80 ± 9	43	42	15	2017	Brazil	Avelino-Silva T.J.et al. PLoS medicine 2017;14:e1002264
Patients with end-stage renal disease	47	69.7 ± 8.95	2	28	70	2018	Poland	Rogowski L. Et al. Adv Clin Exp Med 2018;27:1117–1123
Patients staying on the internal medicine ward Women	76	65 ± 9.7	3	29	68	2018	Poland	Gofąbek K. et al. Rocz Panstw Zakl Hig 2018;69:281–288
Men	44	63 ± 8.4	0	18	82			
Patients hospitalized for a hip fracture	62	79.9 ± 7.7	9	46	44	2018	Italy	Valentini A. et al. Clin Interv Aging 2018;13:1237–1244
Caucasian patients undergoing major surgery	50	73.5 ± 7.76	10	44	46	2018	Italy	Mignini E.V. et al. Eur Rev Med Pharmacol Sci. 2018;22(11):3524–3533
Oncology patients.	296	58.3 ± 11.6	16	44	40	2018	Turkey	Koc E.M. et al. Fam Pract Palliat Care2018;3:39–44
Patients with GIS cancer	153	70.5 ± 5.6	38	35	28	2018	Turkey	Bicakli D.H. et al. Nutrition 2018;47:39–42
Elderly undergoing elective total hip arthroplasty at hospital admission	26	75 ± 1	0	0	100	2019	The Netherlands	Kouw I.W.K. et al. J Am Med Dir Assoc 2019;20(1):35–42
Consecutively admitted internal medicine patients	425	81.2±5.9	23	35	41	2019	China	Miao J.-P. et al. BMJ Open. 2019;9(2):e022993
Hospitalized elderly patients	134	68.9 ± 8.4	43	42	16	2019	Malaysia	Abd Aziz N.A.S: et al. Clin Nutr ESPEN 2019;29:77–85
Very old patients admitted to an Orthogeriatric Unit for the treatment of a hip fracture	150	87.6 ± 4.9	13	49	38	2019	Spain	Sánchez-Castellano C. et al. Nutr Hosp 2019;36(4):813–818
Outpatients ≥70 years at the Emergency Department of the University Clinical Hospital of Valladolid	288	81.1 ± 6.6	15	55	31	2019	Spain	Bolado Jiménez C. et al. Nutrition 2019;66:142–1466
Hospitalized elderly	124	85.9 ± 5.5	50	50		2020	Belgium	Hammami S. et al. BMC Geriatrics 2020;20(1):144
Patients hospitalised on the geriatric wards of the Department of Internal Medicine	240	83.4 ± 8.1	37	61	2	2019	Germany	Becker L. et al. Sci Rep 2019;9:9064
Consecutive patients with end-stage kidney disease on maintenance haemodialysis	113	67.0 ± 16.1	36	48	16	2020	Belgium	Vanden Wyngaert K. et al. PLoS One 2020;15:e0236816
Hospitalized patients diagnosed with Heart Failure with reduced ejection fraction	120	55 ± 11	9	52	39	2020	Poland	Kalużna-Oleksy M. et al. Nutrients 2020;12:2330
**Hospitalized elderly**			**Under-nourished**	**At risk of malnutrition**	**Well-nourished**			
			**<17**	**17 – 23.5**	**≥24**			
Total	33222	mean	22	44.5	34			
		SE	0.2	0.3	0.3

**Table 9:** MNA® Clinical practice — Frail elderly (Outpatient/Home Care) — Identifying the elderly/adults at risk of malnutrition

			**Nutritional status evaluation[% of subjects]**					
**Setting**	**n**	**Age**	**Under-nourished**	**At risk**	**Well-nourished**	**Pub Year**	**Country**	**Reference**
		**[year]**	**<17**	**17 – 23.5**	**≥24**			
Home care Elderly receiving home nursing care	80	84 ± 6	3	63	35	1999	Sweden	Salettti A et al J Hum.Nutr Diet 1999;12:381–387
Home care	529	78±9	6	46	48	1999	Belgium	Ridder D et al. Nestle Nutr Workshop Ser Clin Perform Programme 1999;1:162
Outpatient Elderly patients visiting the university teaching hospital outpatient clinic	53	80±7	2	23	75	1999	Switzerland	Decrey H et al. Nestle Nutr Workshop Ser Clin Perform Programme 1999;1:163
Community geriatric outpatient clinic	463	70	1	8	91	2000	Israel	Maaravi Y et al. Aging (Milano) 2000;12:173–179
Gerontological internal medicine service	71	>65	22	35	14	2000	France	Fanello et al. Sante Publique 2000 Mar;12(1):83–90
Patients >65 years in general practice, with no acute illness	61	75 (72-79)	0	38	62	2001	Denmark	Beck AM et al. Eur J Clin Nutr 2001;55:1028–33
Home Care, Patients with leg and foot ulcers	70	79	2	32	36	1999	Sweden	Wissing U. et al. Scand.J Caring Sci 1999;13:123–128 J Nutr Health Aging 2001;5:37–42
Follow-up for 4 years	43	+ 4 years	13	58	29	2001		
Outpatient Elderly patients referred to a geriatric outpatient clinic	56		11	48	41	2001	U.K.	Cottee M et al. J Nutr Health Aging 2001;5(1):37–42
Outpatient Elderly lived at home in Tallinn	150	58-86	1	39	60	2002	Estonia	Saava M &amp; al. J Nutr Health Aging 2002;6:93–95
Elderly admitted to municipal care	261	83.8 + 7	23	56	21	2002	Sweden	Christensson et al Eur J Clin Nutr 2002;56:810–818
Domiciliary care clients	173	67 – 99	5	38	57	2003	Australia	Visvanathan R &amp; al. J Am Geriatr Soc 2003;51:1007–1011
Frail elderly receiving support services	51	83.7 ± 4.4 (76-93)	1	47	52	2003	Finland	Soini H. &amp; al. Spec Care Dentist 2003;23(6): 209-215
Elderly living at home, receiving Meals-on-Wheels (MOW)	324	60-90	24	65	11	2003	U.S.A.	Kretser AJ. &amp; al J Am Diet Assoc 2003;103:329–336
Municipal home-care services in rural Finland.	178	>75 [75-94)	3	48	49	2004	Finland	Soini H. &amp; al. Eur J Clin Nutr 2004;58:64–70
Home living elderly Swedish women	351	∼73	0	7	92	2004	Sweden	Salminen H. &amp; al Osteoporos Int 2004;15(Suppl1):S52
Outpatients	215	>60	4	31	65	2004	Turkei	Sakarya M. &amp; al Anasthesiol Intensivmed Notfal 2004;39:400–405
Home-care Patients	104	>65	20	52	28	2004	Spain	Ricart Casas J. Aten Primaria 2004;34:238–243
Elderly in various settings was carried out.	226	78.6 + 0.5	20	58	22	2005	Japan	Kuzuya M. et al. Nutrition 2005;21:498–503
Elderly living at home in 5 Swedish municipalities	353	82± 7	8	41	51	2005	Sweden	Saletti A. &amp; al. Gerontology 2005;51:192–198
Patients living at home and receiving home health care services	51	76-93	0	47	53	2005	Finland	Soini H. &amp; al. J Nutr health Aging 2005;9:249–253
Community-dwelling and frail elderly	187	>60	5	50	44.4	2005	South Africa	Charlton K.E. et al. Public Health Nutr 2005;8:468–479
Apartment residents	67	70 ± 2.5	0	34	66	2005	Canada	Lawrence H.P. et al. Spec Care Dentist 2005;25(5):242–252
Elderly service flat residents	80	85.5 (79 – 90)	30	59	11	2005	Sweden	Ödlund Olin A. et al. Eur J Clin Nutr 2005;59:263–270
Residential homes	127	>65	20	50	30	2006	Sweden	Wikby K. et al. J Nutr health Aging 2006;10:232–238
Day-care centers	281	81.9 ± 7.2	9	51	40	2006	Japan	Izawa S. et al. Clin Nutr 2006;25:962–967
Osteoporosis study women	120	69 (60-80)	34	4	62	2006	Lithuania	Ožeraitienė V. &amp; Butėnaitė V. Medicina (Kaunas) 2006;42(10):836–842
Home care patients	178	83.5 ±4.63	3	48	49	2006	Finland	Soini H. et al. J Gerontol Nurs 2006;32(4):12–17
11 Centers of Health	1605	65 – 100)	6	34	60	2007	Spain	Jiménez Sanz M. et al. Nutr Hosp 2007;22(Supl 1):9(Abstr)
Elderly outpatients with and without venous ulcers	77	73.9 ± 8.5	9	39	52	2008	Poland	Szewczyk M.T. et al. Ostomy Wound Manage 2008;54(9):34–6,38-40,42
Ambulatory rehabilitation	229	72 (70, 74)	5	58	37	2008	Australia	Kaur S. et al. Asia Pac J Clin Nutr 2008;17(2):199–207
Frail elderly service flat residents	49	84 (79 – 90)	27	63	10	2008	Sweden	Ödlund Olin A. et al. J Nutr Health Aging 2008;12(5)295-301
Patients with Parkinson's disease	61	72.1 ± 12.5	0	23	77	2008	Italy	Barichella M. et al. Nutr Neuroscience 2008;11(3):128–134
COPD patients attendingoutpatient clinic	32	72 ± 6	0	44	56	2008	Italy	Scichilone N. et al. Age Ageing 2008;37:214–217
Ambulatory patients, Geriatric day hospitals	182	79	3	53	44	2008	Canada	Chevalier S. et al. J Nutr Health Aging2008;12 (10):721–726
Elderly patients admitted to the Geriatrics Outpatient Clinic	413	75 ± 7	13	31	56	2010	Turkey	Saka B. et al. Clin Nutr 2010;29:745–748
Patients aged 60 years and above who presented consecutively at the general outpatient department	500	66.7 ± 6.6	8	12	80	2011	Nigeria	Adebusoye L.A. et al. S Afr Fam Pract. 2011;53(4):355–360 J Nutr Gerontol Geriatr. 2012;31(1):71–85
Cancer patients aged ≥75 years seen at geriatric consultations for final therapeutic decision	161	82.4 (73-97)	25	40	35	2011	France	Chaïbi P. et al. Crit Rev Oncol Hematol 2011;79:302–307
Elderly meal recipients	31	?	6	65	29	2012	Albania	Gray A. et al. J Acad Nutr Diet 2016;112:A95
Elderly people (from two Czech home care agencies and two Slovak home care agencies)	120	73.24	35	33	32	2012	Czech Republic	Kozakova, R. et al. Biomed Pap Med Fac Univ Palacky Olomouc Czech Repub. 2012;156(4):371–376
Elderly from out-patient day hospital and short term rehabilitation	35	79.1 ± 6.8	3	26	71	2012	Ireland	Claesson M.J. et al. Nature 2012:488:178–184
Elderly women at four private care homes in Jakarta	100	72.4 ± 8.2	2	57	41	2013	Indonesia	Adiatman M. et al. Gerodontology 2013;30:262–269
Outpatients with systolic heart failure	50	74.3 ± 6.2	6	10	84	2013	Portugal	Sargento L. et al. J Nutr Health Aging 2013;17:300–3044
Voluntary older home care receivers recruited in the areas of Paderborn, Bonn and Nuremberg	296	80.7 ± 7.7	12	57	31	2013	Germany	Kiesswetter E. et al. J Nutr Health Aging 2013;17:345–350
Patients visiting a geriatric outpatient department	448	80 ± 7	17	58	25	2013	The Netherlands	van Bokhorst-de van der Schueren M-A.E. et al. Clin Nutr 2013;32:1007–1011
Patients of the gastroenterology outpatient department of the Maastricht University Medical Center, a regional referral center for Chronic pancreatitis	50	57.1 (20–81 years)	10	40	50	2013	The Netherlands	Verhaegh B.P.M. et al. Eur J Clin Nutr 2013;67(12):1271–1276
Geriatric day hospital of a large community hospital	190	82 (80-86)	6	45	50	2014	Germany	Schrader E. et al. J Nutr Health Aging 2014;18(3):257–263
Community-dwelling older adults receiving home care	309	80.9 ± 7.9	14	58	29	2014	Germany	Kiesswetter E. et al. J Am Geriatr Soc 2014;62:512–517
Geriatric day hospital unit of the Toulouse Gérontopôle	1108	82.9 ± 6.1	8	40	53	2014	France	Tavassoli N. et al. Nutr Health Aging 2014;18(5):457–464
Respondents who receive nursing care at home	470	77.3	19.2	38.5	42.3	2014	Czech Republic	Kozáková R. et al. Eur Geriatr Med 2014;5:377–381
Elderly of external consult in a Public Specialized Hospital of México City	96	80.4 ± 0.9	11.3	72.2	15.5	2014	Mexico	Perez Cruz E. et al. Nutr Hosp 2014;29:901–906
Internal medicine and geriatrics outpatient clinics	1030	>65	19	29	52	2015	Turkey	Gündüz E. et al. Med Sci Monit 2015; 21: 2750-2756
Patients aged ≥65 years who were admitted to our geriatric medicine outpatient clinic	236	76.4 ± 7.2	15	30	55	2015	Turkey	Sarikaya D. et al. Arch Gerontol Geriatr 2015;61:56–60
Postmenopausal women, outpatient clinics in Ain Shams University hospital	200	45 – 64	11	27	62	2016	Egypt	Gabal H. et al. Eur J Public Health 2016;25:ckv176.300
Patients aged ≥65 years of a geriatric day hospital of a large community hospital in Nuremberg	190	80 (75-84)	6	45	50	2016	Germany	Schrader E.et al. J Nutr Health Aging 2016;20:918–926
Elderly, aged ≥65 years, being treated in community healthcare services in the Community of Madrid: DREAM + 65 Study	1103	79.5 ± 8.4	10	23	67	2016	Spain	Cuerda C. et al. Nutr Hosp 2016;33:263–269
Patients referred to the Geriatric Frailty Clinic.	1309	82.5 ± 6.3	5	35	61	2017	France	Rapp L. et al. J Frailty Aging 2017;6:154–60
Frail elderlyl, home care	80	83 ± 8	9	39	53	2017	Austria	Haider S. et al. PLoS One. 2017;12(1): e0169613
Aged-care facilities in metropolitan Melbourne and regional Victoria	215	85-8 ±7-5	11	57	32	2017	Australia	Iuliano S. et al. Br J Nutr 2017;117:142–147
Patients who visited the geriatric outpatient department of a Dutch hospital	404	80.2 ±7.1	15	56	29	2018	The Netherlands	Kurkcu M. et al. Clin Nutr ESPEN 2018;23:112–116
Elderly outpatients (≥65 years) admitted to a CGA center	473	80.9 ± 6.6	15	34	51	2018	Italy	Liguori I et al. Nutr Clin Pract 2018;33(6):879–886
Outpatients evaluated at the department of internal medicine	50	78.1 ± 6.0	0	22	78	2018	Italy	Valentini A. et al. Clin Interv Aging 2018;13:1237–1244
Elderly patients at a Geriatric Outpatient Clinic	159	76.9 + 6.4	8	33	60	2018	Thailand	Pengsorn N. et al. J Med Assoc Thai 2018:101:869–874
Outpatients with chronic stroke	59	71 ±14.7	0	25	75	2019	Taiwan	Lin S-C. et al. PLoS One 2019;14:e0218749
Secondary outpatient clinic								
Non-Frail	39	73.6 ± 5.7	0	13	87	2019	Brazil	Zukeran M.S. et al. J Nutr Health Aging 2019;23(2):217–220
Pre-frail	76	75.3 ± 7.2	2	31	68			
Frail	139	78.9 ± 7.6	8	54	38			
Elderly over 65 years of age in 3 health centres and 3 residential care homes in Cordoba	248	81.3 (80 – 82)	9	29	62	2019	Spain	Muñoz Díaz B. et al. Family Practice 2019;36:172–178
Elderly home care patients	209	≥ 19	53	30	17	2019	Turkey	Adıgüzel E. &amp; Acar-Tek N. Exp Gerontol 2019;120:15–20
Older patients undergoing cancer care	454	78(65 – 96)	30	35	35	2019	U.S.A.	Zhang X. et al. J Geriatr Oncol 2019;10:763–769 BMJ Supportive &amp; Palliative Care 2020;10:363–368
Patients ≥ 60 years followed in the Geriatric Outpatient Clinic	76	71 (67 – 77)	0	29	71	2020	Poland	Fatyga P. et al. Eur Geriatr Med 2020;11(3):383–391
Dialysis patients of the hemodialysis units	216	67±15	38	43	19	2020	Belgium	Holvoet E. et al. PLoS One 2020;15(3):e0229722
Patients admitted to dedicated rehabilitation wards	1430	79 (74–84)	21	53	26	2020	Australia	Lambert K. et al. J Nutr Gerontol Geriatr 2020;39:16–29
Elderly attending a Primary Care Community Health Center in Guayaquil	196	70.9 ± 7.1	6	52	42	2020	Ecuador	Álvarez Córdova L.R. et al. Nutr Hosp 2020;37:926–932
Outpatients, aged ≥65 years, who applied to a geriatric center between 2017 and 2018	1000	74.3 ± 8.3	7	32	62	2020	Turkey	Kalan U. et al. Aging Clin Exp Res 32:673–680
**Frail elderly Outpatient/Home Care**			**Under-nourished**	**At risk of malnutrition**	**Well-nourished**			
			**<17**	**17 – 23.5**	**≥24**			
Total	19910		11	40	48			
			0.2	0.3	0.4

**Table 10:** MNA® Clinical practice: elderly — Institution — Identifying the elderly/adults at risk of malnutrition

			**Nutritional status evaluation[% of subjects]**						
**Setting**	**n**	**Age**	**Under-nourished**		**At risk**	**Well-nourished**	**Pub Year**	**Country**	**Reference**
		**[year]**	**<17**		**17 – 23.5**	**≥24**			
Medical nursing facility	77	86 + 9	32		43	25	1999	France	Menecier P et al. Age &amp; Nutrition 1999;10:3–6
Residential home residents	100	> 65	5		41	54	1999	Slovakia	Hrabinská L et al. Nestle Nutr Workshop Ser. Clin Perform Programme 1999;1:169
Retirement homes residents	107	65 – 104	21		62	17	1999	Poland	Adamska-Skula M &amp; Lutynsky R Nestle Nutr Workshop Ser. Clin Perform Programme 1999;1:169
Nursing home	100	85 + 9	21		60	19	1999	Italy	Molaschi M et al. Nestle Nutr Workshop Ser. Clin Perform Programme 1999;1:159
Nursing home elderly with dementia	51	86 ± 8	41		45	14	1999	France	Lauque S et al Revue Geriatr 1999;24:115–119
Nursing homeelderly without dementia	24	90 ± 4	21		42	38			
Community setting Elderly admitted from home	261	84 ± 7	23		56	21	1999	Sweden	Christensson L. et al. J Nutr Health Aging 1999;3:133–139
Nursing home	87	82 ± 8	6		47	47	1999	Spain	Salvà A et al. Nestle Nutr Workshop Ser.Clin Perform. Programme 1999;1:123–129
Retirement homes	81	83 + 7 (61-98)	2		37	61	2000	Belgium	Griep MI et al. J Gerontol A Biol Sci Med Sci 2000;55:M57-M63
Long term care	431	>60	71		26	4	2000	Italy	Donini LM et al. Age &amp; Nutrition 2000;11:2–5
Long term care	77	85.6 ± 8.8	32		43	25	2000	France	Menecier-Ossia L. et al. Revue Geriatr 2000;25:65–70
Institution (all)	872	84.5 ± 8	36		48	16	2000	Sweden	Saletti A et al. Gerontology 2000;46:139–145
Rehabilitation unit	73	80.4 ± 7.6	23		67	10	2000	Switzerland	Liver C et al. Age &amp; Nutrition 2000;11:67–71
Institutionalized elderly Chinese	120	>60	21		52	26	2001	China	Hui WH. et al. Hong Kong J Gerontol 2001;15:35–43
Nursing home	150	58–96	1		27	73	2002	Estonia	Saava M. &amp; Kisper-Hint I.-R. J Nutr Health Aging2002; 6:93–95
Spanish institution									
Women	134	>65	5		38	58	2001	Spain	Ramon J.M. &amp; al Med Clin (Barc) 2001;117: 766-770
Men	255		9		46	45			
Nursing home	66	>65	32		55	9	2002	Denmark	Beck AM. &amp; al Aging Clin Exp Res. 14:212–215, 2002
Municipal care	261	65–107	23		56	21	2002	Sweden	Christensson et al. Eur J Clin Nutr 2002;56:810–818
Long term geriatric unit Mataró	67	83 ± 8	24		37	39	2002	Spain	Bleda MN &amp; al J Nutr Health Aging 2002;6:134–37
Nursing homes									
Madrid	205	>65	35		51	24	2002	Spain	Ribeira Casado J.M. J Nutr Health Aging 6:84–90, 2002 Sánchez, R. Residential 1999;9:21–30. Latorre C. et al. Rev Esp Geriatr Gerontol 2000;35(supl 1):74–75
Valencia	94	79.3	5		45	50			
Institutionalized patients with a diagnosis of AD according with NINCDS/ADRDA criteria from 8 nursing-homes	99	86.5 ± 6.1	17		68	14	2003	Spain	Gregorio P.G. &amp; al J Nutr Health Aging 2003;(7)5:304–308
Institutionalized older women	89	85 ± 6 (72-98)	8		82	30	2003	Spain	Ruiz-López M.D. et al. Nutrition 2003;19:767–771
Nursing homes residents	90	86 ± 6	13		63	23	2003	Switzerland	Gerber V. et al. J Nutr Health Aging 2003;7:140–145
Nursing Home elders with pressure ulcers	24	>65	54		29	17	2004	USA	Hudgens JH &amp; al. JPEN J Parenter Enteral Nutr 2004;28(6):416–422
Elderly from a geriatric home	63	>60	14		70	16	2004	Venezuela	Pena E. et al. Rev Esp Geriatr Gerontol 2004;39(6):360–366
Patients admitted at our subacute care nursing home	352	81.5 ± 8	38		55	7	2004	Italy	Baldelli M.V. et al. Arch Gerontol Geriatr Suppl 2004;(9):39–43
Nursing home residents	50	81.3	16		54	30	2004	Spain	Villaverde Gutierrez C. et al. Geriatrika 2004;20(1):8–11
Two municipal service flat complexes	80	79-90	30		59	11	2005	Sweden	Odlund Olin A. et al. Eur J Clin Nutr 2005;59(2): 263-270
All nursing homes in Helsinki community Women	1696	84 ± 8.5	30		60	9	2005	Finland	Suominen M. &amp; al. Eur J Clin Nutr 2005;59: 578–583 Eur J Clin Nutr 2009;63:292–296
Men	409	80 ± 8.5	23		61	17			
Subjects from residential homes	237	83.2 ± 8.8	5		60	35	2005	Italy	Cairella G. et al Ann Ig 2005;17:35–46
Elderly institutionalzed patients	153	76.9 ± 9.7	19		46	36	2005	Brazil	Alves de Rezende C.H. et al. Gerontol 2005;51:316–321
Nursing home &amp; chronic care	31	65 ± 2.8	7		48	45	2005	Canada	Lawrence H.P. et al. Spec Care Dentist 2005;25(5):242–252
Institutionalized elders in different geriatric units of the metropolitanarea Caracas	126	60 – 96	6		46	46	2005	Venezuela	Rodriguez N. et al. Invest Clin 2005;46:219–228
Nursing home	178	77	14		64	23	2006	The Netherlands	Nijs K.A.N.D. et al. J Gerontol 2006;61A(9):935–942
Older residents of the nursing home for the somatically ill adults in Bialystok	100	79.1 ±7.8	12		61	27	2006	Poland	Wojszel Z.B. Adv Med Sci. 2006;51:168–173
Elderly persons living in 5 nursing homes, Tyrol	272	84.4 ± 8.9	19		50	32	2006	Austria	Jeske M. et al. Journal fur Ernährungsmedizin 2006;8(1):13–20
100 institutions, rhône-alpes region	687	85 ± 8.16 (60-110)	18		41	41	2007	France	Dion N. et al. Nutrition 2007;23:301–307
Homes for the aged, Tygerberg Academic Hospital	210	76.8 ± 10.6	6		47	47	2007	South Africa	Marais M.L. et al. South Afr J Clin Nutr. 2007;20(3):102–108
Elderly nursing home	145	81 ± 7	3		35	61	2007	Japan	Shimizu T. et al. Ann Nutr Metab 2007;51:413 Abstr 11
Nursing home residents	112	85 (79 – 91)	9		71	20	2007	Germany	Norman K. et al. Nutrition 2007;23:564–569
Nursing home residents	83	85	0		29	71	2007	The Netherlands	Essed N.J. et al. Appetite 2007;48:29–36
Long-term residential care establishment High-level &amp; Low-level Care facilities	75	>65	16		37	47	2007	Australia	Grieger J.A. &amp; Nowson C.A. Eur J Clin Nutr 2007;61:655–663
Patients admitted to internal medicine departments	589	>65	8		36	56	2007	Israel	Feldblum I. e tal. Nutrition J 2007;6:37
Institutionalized elderly, Nursing homes from the Granada province	82	83,1 ± 5,6	19		11	70	2007	Spain	Pérez Moreno A. et al. Nutr Hosp 2007;22(supl 1):92(Abstr)
Residents of long-term homes for the elderly, São Paulo	40	≥ 60	10		40	50	2007	Brazil	Santelle O. et al. Cad Saúde Publica 2007;23(12):3061–3067
Nursing home residents	106		50		45	5	2007	Cuba	González Hernández A. et al. Arch Latinoam Nutr 2007;57:266–272
Long term care centre in central Taiwan: Cognition-normal elderly	169	79.6 ± 7.6	22		59	19	2008	Taiwan	Tsai A.C. &amp; Ku p-Y. Br J Nutr 2008;100(1):152–15
Institutionalized elderly in a nursing home	50	84 (66 – 97)	6		12	82	2008	Spain	Abajo del Alamo C. et al. Nutr Hosp 2008;23(2):100–104)
Elderly from long-term institution in southeast	89	73.7 ± 9.1	28		51	21	2008	Brazil	Ferreira L.S. et al. J Nutr Health Aging 2008;12(3):213–217
Large Vienna nursing home	245	86 ± 7	38		48	14	2008	Austria	Kulnik D. &amp; Elmadfa I. Ann Nutr Metab 52(Suppl 1):51–53, 2008
Community resident homes	127	women 86 ± 6.0 men 84 ± 6.8	16		53	31	2008	Sweden	Wikby K. et al. J Clin Nursing 2008;17:1211–1218
Long-term care resident elderly	172	85.3 ± 8.4	20		51	29	2008	Italy	Cereda E. et al. Clin Nutr 2008;27:700–705
Institutions South-West: Nursing-home residents	517	84.6 ± 9.0	13		42	45	2009	France	Bourdel-Marchasson I. et al. Nutrition 2009;25:155–164
Nstitutions South-West: Long-term care home residents	84	81.8 ± 10.4	43		48	10			
Nursing home residents	43	82.5 (81;85)	10		72	19	2008	Malta	Koh G.C. Malta Med J 2008;17(01):28–42
Nursing home residents	50	84 (66 – 97)	6		12	82	2008	Spain	Abajo del Alamo C. et al. Nutr Hosp 2008;23(2):100–104
Long Term care stroke patients	74	>40	24		57	19	2008	Taiwan	Tsai A.C. &amp; Shih C-L. J Clin Nursing 2008;18:82–88
Nursing home residents	199	83 ± 8	40		56	5	2009	Finland	Kuikka L.K. et al J Am Med Dir Assoc 2009;10:348–353
Geriatric homes in Cairo	100	>60	28		22	50	2010	Egypt	Amer M.S. et al. J Am Geriatr Soc 2010;58(10):2036
Veterans' Administration Hospital-managed nursing home in Central Taiwan	160	81.1	19		54	26	2010	Taiwan	Tsai A.C. et al. Arch Gerontol Geriatr 2012;54(3):443–447
Kahrizak Charity Foundation (KCF) residents	221	78.1 ± 7.5	3		43	53	2010	Iran	Amirkalali B. et al. Public Health Nutr. 2010;13(9):1373–1379
Nstitutionalized elderly aged over 60 years	344	75.4 ± 9.4	8		56	36	2011	Brazil	Pereira Machado R &amp; Coelho M. J Nutr Health Aging 2011;15:532–535
Residents of three nursing homes in Cairo	120	71.4 ± 6.9	11		41	48	2011	Egypt	Khater M.S. et al. J Nutr Health Aging. 2011;15(2):104–108 J Clin Gerontol Geriatr. 2012;3(2):73–76
Residents from 3 municipal nursing homes in Bonn	350	84.8 ± 8.0	27		53	20	2011	Germany	Volkert D. et al. Gastroenterol Res Pract 2011;2011:247315
Elderly residing in long-term residential care	57	83.5 ± 7.6	47		49	4	2012	Ireland	Claesson M.J. et al. Nature 2012:488:178–184
Institutionalised elderly population in Mysore city,	141	72.2 ± 7.5	15,6		53	32	2013	India	Kshetrimayum, N. et al. Gerodontology 2013;30:119–125
Residents of retirement homes in and around Prague							2013	Czech Republic	
Women	659	86.1 ± 6.2	11		41	48			Rambousková J. et al. Ann Nutr Metab 2013;62:201–206
Men	156	81.5 ± 8.0	8		32	70			
Resident patients in the nursing home facility	100	80.2± 10	36		46	18	2013	Italy	Donini L.M. et al. PLoS ONE 2013;8(2):e55804
Subjects admitted in the nursing homes									
women	195	81.6± 8	43		43	14	2013	Italy	Donini L.M. et al. J Nutr Health Aging 2013;17:332–328
Men	121	77.5± 8	31		35	35			
Elderly ≥ 65 years, living consistently in the nursing home, and not in final stage of life (ernstes study)	650	85 (81, 91)	10		48	42	2013	Germany	Strathmann S. et al. J Nutr Health Aging 2013;17:271–276
Residents of 6 German nursing homes	286	86± 7	18		42	40	2013	Germany	Stange I. et al. J Nutr Health Aging 2013;17:357–363
Two municipal nursing homes in Nuremberg	188	85.5± 7.8	15		57	27	2013	Germany	Diekmann, R. et al. J Nutr Health Aging 2013;17:326–331
Elders living in 34 nursing homes all over the province of Albacete									
Women	523	82.9± 6.7	4		40	56	2013	Spain	Serrano-Urrea R. et al. Gerontology 2013;59:490–498
Men	372	81.3± 7.6	2		33	65			
Elderly institutionalized in a nursing home	36	85.7	33		58	8	2013	Spain	Calvo M.E.D. Rev Esp Nutr Comunitaria 2013;19:20–28
Institutional environment (nursing homes)	859	79.0± 7.9	16		49	35	2014	Poland	Kostka J. et al. J Nutr Health Aging 2014;18:366–371 Eur J Clin Nutr 2014;68:1210–1215
15 Nursing Homes in the city of Salvador	359	74.3± 8.7		66		34	2014	Brazil	Amorim Sena Pereira M. et al. Nutr Hosp 2014;31:1198–1204
Public long term geriatric units	344	75.4± 9.4	8		56	36	2015	Brazil	Machado R.S.P. et al. BMC Geriatr. 2015;15:132
Residential homes for the elderly in Lattakia	103	70.9± 6.4	19		40	41	2015	Syria	Hallaj F.A. East Mediterr Health J. 2015;21(10):753–761
Institutionalized elderly, aged ≥ 65 years, in 25 institutions in 19 cities throughout Turkey.	554	76.1± 7.3	7		49	44	2015	Turkey	Ongan D. and Rakıcıoğlu N. Arch Gerontol Geriatr 2015;61:271–276
Nursing home residents									
Women	164	82.3± 9	22,6		57	21	2016	Italy	Donini L.M. et al. J Am Med Dir Assoc 2018;17(10):959. e11-959.e18
Men	82	76.5± 11	17		61	22			
Senior citizens living at old age homes of Kathmandu valley	213	77.2± 8.7	16		61	24	2016	Nepal	Singh D.R. et al. Int J Community Med Public Health. 2016;3(7):1707–1715 Gait Posture 2017;54:56–61
Long-stay nursing home residents	123	82.7 ± 9.0	18		54	29	2016	Portugal	Pinho J. et al. Clin Nutr 2016;35(Suppl 1):S108
Older adults residing in care homes	97	82.2 ± 6.3	3		27	70	2016	Portugal	Araújo D.A. et al. Eur J Clin Nutr 2016;70:859–862
Elderly residing in long-term care facilities from urban Bloemfontein									
lower socio-economic area	62	78 (58–99)	11		74	15	2017	South Africa	Robb L.et al. South Afr J Clin Nutr 2017;30:34–40
Higher socio-economic area	62	85(62–95)	3		37	60			
Homes, shelters, and care centers for the elderly	144	75.3	11		82	7	2018	Ecuador	Espinosa Del Pozo P.H: et al. Cureus 2018;10(9):e3269
Elderly denture-wearing patients above 60 years	200	> 60	20		70	11	2018	India	Banerjee, R. et al. Indian J Dent Res. 2018;29:562567
Institutionalized elderly from North Bohemia									
Women	183	80.8 ± 8.1	12		52	36	2018	Czech Republic	Slavíková M. et al. Cent Eur J Public Health 2018; 26 (2):111–117
Men	71	75.9 ± 8.7	7		45	48			
Frail elders, aged 75 years or over from two nursing homes	81	≥75	0		40	61	2018	Spain	González I. et al. Proceedings 2018;2:1247
A socio-sanitary residence in the region of El Bierzo	164	85.6 ± 7.54)	35		42	24	2019	Spain	Penacho Lázaro M.Á. Et al Nutr Hosp. 2019;36(2):296–302
Elderly ≥ 60 years in 4 homes for the elderly in Bogotá	152	81.5 ± 7.8	6		34	60	2019	Chile	Díaz-Muñoz G.A. and Calvera-Millán S.J. Rev Chil Nutr 2019;46:746–752
Malatya nursing home	65	77.4 ± 8.7	15		42	43	2019	Turkey	Bentli R. et al. Medecine Sci. 2029;8(2):430–435
Nationally representative sample of the Portuguese population aged 65 years or over living in nursing homes: PEN-3S-Study	1186	83.4	5		39	46	2019	Portugal	Madeira T. et al. Public Health Nutr 2019;22(3):486–497 Nutrition 2020;73:110660
Institutionalised elderly patients in a public nursing home	86	78.6 (53–101)	17		34	49	2020	Spain	Puivecino Moreno C. et al. Eur J Hosp Pharm Sci Pract 2020;27:A143
3 nursing homes and 14 assisted living facilities in Helsinki									
Prefrail	148	≥ 65	6		66	28	2020	Finland	Salminen, K. S. et al. J Nutr Health Aging 2020;24(3):319–324
Frail	228	≥ 65	20		68	12			
Elderly population living in Old Homes of Jodhpur and Pali District, desert areas of Rajasthan	158	≥ 60	12		44.3	42.7	2020	India	Vyas K. &amp; Singh M. Int J Res Anal Rev 2020;6:i48-i50
Older People Living in the Community	100	74.9 ± 8.50	14		63	23.0	2020	Poland	Piglowska M. et al. Nutrients 202; 12(7): 2042
**Institutionalized elderly**			**Under-nourished**		**At risk of malnutrition**	**Well-nourished**			
			**<17**		**17 – 23.5**	**≥24**			
Total	23119	Mean	18		48	34			
		SE	0.3		0.3	0.3

**Table 11:** MNA® Clinical practice: elderly — Cognitively impaired elderly — Identifying the elderly/adults at risk of malnutrition

			**Nutritional status evaluation[% of subjects]**					
**Setting**	**n**	**Age**	**Under-nourished**	**At risk**	**Well-nourished**	**Pub Year**	**Country**	**Reference**
		**[year]**	**<17**	**17 – 23.5**	**≥24**			
Elderly subjects with dementia in a nursing home	51	86.2 ± 7.5	41	45	14	1999	France	Lauque S et al. Revue Gériatrie 1999;24:115–119
Psychogeriatric hospitalday hospital patients	133	75 ± 7	14	54	32	1999	Switzerland	De Mendonca Lima CA et al. Age Nutr 10:9–13, 2001
Non-institutionalized women with sarcopenia and Alzheimer's disease	32	81.5 ± 4.9	29	43	29	2000	France	Gillette-Guyonnet S et al. J Nutr Health Aging 200;4(3):165–169
Home living Azheimer'sdisease elderly patients	100	76 + 12	6	36	58	2001	France	Rivière S et al. J Nutr Health Aging 2001;5:295–299
Home living Alzheimer's disease patients (ELSA study)	318	75(45-89)	1	19	80	2001	France	Andrieu S et al. J Nutr Health Aging 2001;5:113–117 Dumont C. Et al. J Nutr Health Aging2005;9:163–167
Memory Clinic Community dwelling subjects referred to a memory clinic	123	75 ± 7	2	33	64	2002	Ireland	Fallon C et al. J Nutr Health Aging 2002;6(Suppl):21
Demented patients admitted to an Alzheimer section	174	80.2 ± 8.1	36	48	17	2003	Italy	Magri F. &amp; al Aging Clin Exp Res 2003;15(2):148–153
REAL.FR, Alzheimer's disease	479	77.4 + 7.1	5	35	61	2003	France	Brocker P. &amp; al Rev Med Interne 2003:24:314S-318S Gillette-Guyonnet S. Et al. J Nutr Health Aging 2003;7(2):91–96
Elderly with perceived imaired memory	59	74.3 (52-86)	14	63	24	2003	Sweden	Holm B. &amp; Söderholm O. Clin Nutr 2003;22(4):385–389
Nursing home residents with dementia	23	69 – 89	13	87	0	2004	Sweden	Suominen M. &amp; al J Nutr Health Aging 2004;8(4):234–238
Cognitive impaired patients, geriatric convalescence unit	63	80.1+8.1	62	37	2	2004	Spain	Arellano M. &amp; al Arch Gerontol. Geriatr. Suppl. 2004;9:27–31
AD patients living at home (REAL.FR Study)	561	76 + 6	3	18	79	2005	France	Guérin O. Et al. J Nutr Health Aging 2005;9:75–80 Vellas B. Et al. J Nutr Health Aging 2005;9(2):81–84
Patients with Alzheimer's disease (ELSA Study)	312	75.4 ± 6.7	1	22	77	2005	France	Dumont Ch. Et al. J Nutr Health Aging 2005;9:163–167)
Long term care centre in central Taiwan: Cognition-impaired elderly	139	82.2 + 7.9	34	54	12	2007	Taiwan	Tsai A.C. &amp; Ku p-Y. Br J Nutr 2008;100(1):152–158
Very mild Alzheimer disease patients	160	75.7± 5.8	0	28	72	2008	France	Ousset P-J et al. Alzheimer Dis Assoc Disord 2008;22:66–71
Elderly, diagnosed with probable or possible Alzheimer's disease, living at home with a well identified informal caregiver (PLASA study	562	79.6 ± 5.7	0	36	64	2008	France	Nourhashemi F. Et al. J Nutr Heath Aging 12(4):263–271, 2008
Patients entering the severe stage of AD in the longitudinal study of REAL.FR cohort	126	78.5 ± 8.1	9	60	32	2009	France	Gillioz A.S. et al. Dement Geriatr Cogn Disord 2009;28:427–32
Hospitalized elderly patients with mild cognitive impairment	623		24	58	18	2009	Italy	Orsitto G. Et al. Clin Nutr 2009;28:100–102
Patients diagnosed with Alzheimer's disease with advanced cognitive impairment	52	84.5 ± 7.1	35	48	17	2009	Spain	Tarazona Santabalbina F. J. Et al. Nutr Hosp. 2009;24:724–731
Alzheimer's Disease Unit	49	73.9 ± 7.4	0	43	57	2010	Italy	Spaccavento S. Et al. Arch Gerontol Geriatr. 2009;48(3):356–360
Residents with mild cognitive impairment of three nursing homes in Cairo	46	74.5 ± 6.7	17	54	28	2011	Egypt	Khater M.S. et al. J Nutr Health Aging. 2011;15(2):104–108 J. Clin. Gerontol. Geriatr 212;3:73–76
Community-dwelling older people with dementia	56	80.7 (SD 6.5)	23	59	18	2013	France	Rullier L. Et al. Int J Geriatr Psychiatry. 2013;28(6):580–588
Community-dwelling elderly with newly diagnosed Alzheimer's disease	312	77.6 ± 5.7	0	14	86	2013	The Netherlands	Droogsma E. Et al. J Nutr Health Aging. 2013;17(7):606–610
Community-dwelling individuals attending dementia clinics (nutrialz Study)	940	79.1 ± 7.3	5.2	42.6	52.2	2013	Spain	Roqué M. Et al. J Nutr Health Aging. 2013;17(4):295–299 Salvá A. Et al. J Nutr Health Aging. 2009;13(6):529–537
Memory clinic patients with mild cognitive deficits	48	70.6 (61 – 87)	0	39	61	2013	Germany	Von Arnim C.A.F. et al. Nutr J 2013;12:148
Community-dwelling persons aged 60 years and above, prevalence of dementia 0.151 from 471 elderly	70	60+	40	54	6	2014	Bengladesh	Palmer K. Et al. Int Psychogeriatr 2014;26:1905–1915
Subjects diagnosed with probable Alzheimer's disease	189	82.3 (69-101)	9	55	36	2014	Spain	Sarabia-Cobo C.M. et al. J Aging Res Clin Practice 2014;3(3):178–181
Individuals of the geriatric outpatient department, with memory complaints, and with multiple problems in the somatic, psychological, social, or functional domain	359	80 ± 7	13	55	32	2016	The Netherlands	De van der Schueren M.A.E. et al. J Am Geriatr Soc 2016;64:2457–2463
Acutely ill patients admitted to a geriatric ward of a tertiary university hospital in São Paulo, subgroup with Dementia alone	182	82 ± 8	48	42	10	2017	Brazil	Avelino-Silva T.J.et al. Plos medicine 2017;14:e1002264
Elderly with mild, moderate and severe Alzheimer's disease, neurology outpatient clinic	43	80.6 ±7.0	3	66	31	2018	Brazil	Santos T.N.B. et al. Nutr Hops 2018;35(6):1298–1304
Subjects who lived in their own homes and attended the Cognitive Disorders Unit	111	78.5 ± 6.4	19	68	14	2018	Spain	Rocaspana-García M. Et al. Peer 2018;6:e5150j
Older residents in institutional settings in Helinki, assessed witha clinical dementia rating (CDR)								
very mild/mild dementia (CDR 0.5–1),	150	85 ± 7	9	68	23	2019	Finland	Salminen K.S. et al. Nutrients 2019;11(10):2261
Moderate dementia (CDR 2)	206	84 ± 8	17	69	14			
Severe dementia (CDR 3)	182	83 ± 7	28	67	5			
NUDAD study								
Patients with clinical diagnosis of mild cognitive impairment	135	66.3 ± 7.7	1	19	80	2019	The Netherlands	Doorduijn A.S. et al. Nutrients 2019;11:1161
Alzheimer's disease	198	67.4 ± 7.9	3	28	69			
Control group: subjects with subjective cognitive decline, with memory complaints but normal on all clinical examinations	219	60.6 ± 7.7	1	9	90			
PEN-3S Study Cognitively impaired community-dwelling older adults	185	≥ 65	3	40	58	2020	Portugal	Madeira T. Et al. Nutrition 2020;73:110660
Cognitively impaired nursing home residents	608	≥ 65	5	49	47			
**Cognitively impaired elderly**			**Under-nourished**	**At risk of malnutrition**	**Well-nourished**			
			**<17**	**17 – 23.5**	**≥24**			
Total	8378	Mean	14	45	41			
		SE	0.4	0.5	0.5

**Table 12:** MNA® Clinical practice: elderly/adults with Parkinson's disease — Institution — Identifying the elderly/adults at risk of malnutrition

			**Nutritional status evaluation[% of subjects]**					
**Setting**	**n**	**Age**	**Under-nourished**	**At risk**	**Well-nourished**	**Pub Year**	**Country**	**Reference**
		**[year]**	**<17**	**17 – 23.5**	**≥24**			
Patients with Parkinson's disease	61	70.5 ± 5.5	0	23	77	2008	Italy	Barichella M. Et al. Nutr Neuroscience 2008;11(3):128–134
Outpatients with Parkinson's disease and their respective caregivers	117	65 ± 9.4	2	20	79	2010	China	Wang, G. Et al. Parkinsonism Relat Disord 2010;16:119–123
Community-dwelling adults with Parkinson's disease, aged >18 years	125	70.0 (35–92)	2	22	76	2013	Australia	Sheard J.M. et al. E-SPEN Journal 2013;8:e187-e92
Consecutive patients with idiopathic Parkinson's disease, single referral Movement Disorders Clinic in Tehran	150	61 ± 10.8	2	25	73	2014	Iran	Fereshtehnejad S-M. Et al. Plos One 2014;9:e91153 J Parkinsons Dis 2014;4:473–481
Parkinson's disease (PD) patients	114	66 ± 9.8	7	28	65	2017	Argentina	Bril A. Et al. NPJ Parkinsons Dis 2017;3:17
Patients with idiopathic PD, recruited from an outpatient referral movement disorder clinic	96	64 ± 6.4	25	43	32	2018	Turkey	Ongun N. Plos One 2018;13(10): e0205100
Hospitalized elderly patients with Parkinson's disease [MNA® -SF]	92	74 ± 6.7	7	39	54	2020	Germany	Gruber M.T. et al. Plos One 2020;15:e0232764
**Elderly/adults with Parkinson's disease**			**Under-nourished**	**At risk of malnutrition**	**Well-nourished**			
			**<17**	**17 – 23.5**	**≥24**			
Total	755	Mean	6	29	65			
		SE	0.9	1.6	1.7

**Table 13:** Self-MNA® Clinical practice: elderly — Identifying the elderly/adults at risk of malnutrition

			**Nutritional status evaluation[% of subjects]**					
**Setting**	**n**	**Age**	**Under-nourished**	**At risk**	**Well-nourished**	**Pub Year**	**Country**	**Reference**
		**[year]**	**<8**	**8 – 11**	**≥12**			
Community-dwelling older adults	463	76.8 ± 6.8	27	38	35	2013	U.S.A.	Huhmann M. Et al. J Nutr Health Aging 2013;17:339–344
Older who came for care to the Rutgers School of Dental Medicine (RSDM) clinics	75	71.9 ± 5.5	9	28	63	2016	U.S.A.	Stump M. Et al. J Acad Nutr Diet 2016;116:A94
Patients referring to the GP offices Women	125	75.1 ± 8	9	33	58	2018	Italy	Donini L. Et al. J Nutr Health Aging 2018;22:44–52
Men	101	75.3 ± 8	8	21	70			
Community-dwelling older adults	107	72.6 ± 5.6	5	21	75	2018	U.S.A.	Zelig R. Et al. J Aging Res Clin Pract 2018;7:107–114
**N**	**871**	**Mean**	**11.7**	**28.2**	**60.1**			
		**SE**	**1.1**	**1.5**	**1.7**

**Table 14:** Prevalence of malnutrition and risk of malnutrition in different settings MNA®

			**Mean ± SD in %**		
**# of studies**	**Setting**	**Malnutrition**	**Risk of malnutrition**	**Well nourished**	**N**
88	Community	5	31	64	75396
		0.1	0.2	0.2	
73	Home care / Out patient	11	40	48	20701
		0.2	0.3	0.3	
120	Hospital	22	45	34	33222
		0.2	0.3	0.3	
94	Institutions	18	48	34	23246
		0.3	0.3	0.3	
34	Cognitive impaired	14	45	41	8378
		0.8	0.5	0.5	
7	Elderly/adult with Parkinson's disease	6	29	65	755
		0.9	1.6	1.7	
*416*	*Total n of studies*			*Total n of elderly*	*161698*
32	Community	4	27	69	53617
		0.1	0.0	0.2	
20	Outpatient	12	33	55	8508
		0.3	0.1	0.5	
62	Hospital	29	45	27	31068
		0.3	0.0	0.3	
16	Institution	22	50	28	12618
		0.4	0.1	0.4	
3	Cognitively impaired elderly	20	54	26	1980
2	Elderly/adult with Parkinson's disease	4	35	61	217
*133*	*Total n of studies*			*Total # of elderly*	*108008*

**Figure 2:**
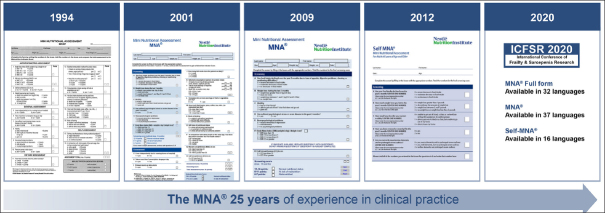
The MNA® forms

The MNA® and MNA®-SF have been validated in many studies, used as reference standard to validate other screening tools and compared to following screening tools: MUST, NRS-2002, GNRI, SGA, PG-SGA, NRI, SNAQ, MST, and NUTRI score, in different settings, community, home care, nursing homes and hospitals (20, 30, 34, 42; 47, 53, 123, 181, 227, 228, 229, 230, 231, 232, 233, 234, 235, 236, 237, 238, 239, 240, 241, 242, 243, 244, 245, 246, 247, 248, 249, 250, 251, 252, 253, 254, 255, 256, 257, 258). The sensitivity and specificity of the MNA® are 80% (SD 13) and 68% (SD 22), respectively, against a wide range of criteria in 40 studies (see table 15: MNA® Sensitivity/Specificity against Nutritional assessment parameters & other parameters) (140, 222, 227, 228, 235, 241, 246, 248, 259, 260, 261, 262, 263, 264, 265, 266, 267, 268, 269, 270, 271, 272, 273, 274, 275, 276, 277, 278, 279, 280, 281, 282, 283, 284, 285, 286, 287). The same is true for the MNA®-SF with a sensitivity of 87% (SD 10) and a specificity of 85% (SD 15) against the MNA®, and a sensitivity of 81% (SD 18) and a specificity of 63% (SD 20) against a wide range of criteria in 43 studies (Tables 16 & 17) (8, 12, 20, 41, 54, 67, 140, 222, 227, 228, 230, 234, 235, 240, 247, 266, 270, 278, 282, 284, 286, 288, 289, 290, 291, 292, 293, 294, 295, 296, 297, 298, 299, 300, 301, 302, 303, 304, 305, 306, 307). In general, it can be observed that MNA®-SF and MNA® are the most used tools to evaluate the risk of malnutrition in the elderly, independent of the setting, with high sensitivity, ≥ 80%, and a good specificity, ≥ 60% (Tables Table 15, Table 16, Table 17), and see meta-analysis/systematic review, reviews, and content validity (1, 43, 47, 149, 182, 221, 308, 309, 310, 311, 312, 313, 314, 315, 316, 317, 318, 319, 320). MNA®-SF and MNA® are appropriate screening and assessment tools for use in community-dwelling elderly (321), and all other geriatric settings (205, 322, 323, 324, 325, 326, 327, 328, 329). Further the MNA®s are the only tools to evaluate the intake of nutrient-rich food groups, which allow for implementation of nutritional intervention. Discrepancies with other screening tools come mainly from the dietary assessment and the difference in weight loss evaluation. It is the only assessment tool assessing two «functional concept» related to muscle and cognitive function. MNA® can be improved by the addition of inflammatory factors or other biological nutritional biomarkers when needed.

**Table 15:** MNA® Sensitivity/Specificity against Nutritional assessment parameters &amp; other parameters

**Reference**	**Sensitivity**	**Specificity**	**References**
Clinical Status (extensive evaluation by 2 clinicians)	96	98	Guigoz Y. Et al. Med Hyg 1995; 53:1965–1969.
Detailed nutritional assessment	54	61	Azad N. Et al. CMAJ 1999;161:511–515
Albumin (<35 g/l)	75	50	
Energy intake (<1 SD mean)			
720 kcal/day	100	37	Murphy M.C. et al. Eur J Clin Nutr 2000;54:555–562
970 kcal/day	72	32	
Mindex (<50%tile 81.7 kg/m)	81	47	
Detailed nutritional assessment (albumin, BMI, diet history, clinical data)	72	88	Hui W.H. et al. Hong Kong J Gerontol 2001;15:35–43
BMI 19	41	86	
BMI 21	59	78	Thomas D.R. et al. Am J Clin Nutr 2002;75:308–313
BMI 22	70	71	
Protein energy malnutrition (weight, triceps skin fold, arm circumference, albumin &amp; transthyretin)	96	26	Christensson L. Et al. Eur J Clin Nutr 2002;56(9):810–818.
Nutritional assessment (anthropometry, serum proteins)	98	13	Donini L.M. et al. J Nutr Health Aging 2002;6:141–146.
Detailed nutritional assessment	90	88	Visvanathan R. Et al. Age Ageing 2004;33:260–265
Nutritional diagnosis (BMI &amp; laboratory testing)	100	74	Delacorte R.R. et al. J Nutr Health Aging 2004; 8:531–534
Full nutritional assessment	77	36	Thorsdottir I. Et al. J Hum Nutr Diet 2005;18:53–60.
PS SGA baseline	97	54	Read J.A. et al. Nutr Cancer 2005;53:51–56.
PG-SGA 4–6 wk	79	69	
PG-SGA 8–12 wk	82	66	
Albumin <3.5 g/dl	86	82	Kuzuya M et al. Nutrition 2005;21:498–503.
NRS-2002	81.7	84.6	Martins C.P.A.L. et al. J Nutr Elderly. 2005;25:5–21
PEM (anthrop., Alb, Prealb)	73	31	Wikby K. Et al. J Nutr Health Aging 2006;10:232–238
Criteria of Edington, Clin Nutr 2000;19:191–195	93	67	Gehring N. Et al. Swis Med Wkly 2006;136:664–669
Fat-free mass index (FFMI; kg/m^2^)	85	39	Elkan A.C. et al. EJCN 2007;62:1239–1247
SGA	77	82	Capra S. Nutrition 2007;23:356–357
Well nourished			
with following nutritional indicators: BMI ≥ 21, Arm Circumference ≥ 22, Calf circumference ≥ 31, albumin ≥ 35	87.1	50	Cuyac L.M. and Santana P.S. (2007)
Arch Latinoam Nutr 2007;57:255–265			
Criteria of the American Institute of Nutrition (AIN): BMI ≤ 20, Arm-circumference ≤ 21 cm, albumin ≤ 3,5 g/dl and cholesterol ≤ 150 mg/dl	60	94.7	Tarazona Santabalbina F.J. et al. Nutr Hosp. 2009;24:724–731
BMI <24 kg/m^2^ Serum albumin <3.5 g/dl	82	63	Amirkalali B. Et al. Public Health Nutr 2010;13:1373–9
SBMI and/or Weight loss: severely undernourished and moderately undernourished versus not undernourished Accuracy for MNA® <23.5	90	36	Kruizenga H.M. et al. J Nutr Health Aging 2010;14:83–89
BMI and/or Weight loss: severely undernourished versus moderately and not undernourished Accuracy for MNA® <17	56	58	
CONUT (controlling nutritional status): albumin, total cholesterol and total lymphocyte count	77	70	Jürschik Jiménez P. Et al. Arch Latinoam Nutr 2009;59(1):38–46
A dietitian assessment was used as the gold standard	80	90	Harris D.G. et al. J Hum Nutr Diet. 2008;21:3–9
SGA	95	61	Velasco C. Et al. Eur J Clin Nutr. 2011;65:269–274
To identify the ability for predicting development of complications in hospitalized patients	73	57	Ocón Bretón M.J. et al. Nutr Hosp 2012;27:701–706
Frailty	56	91	Dent E. Et al. J Nutr Health Aging 2012;16:764–767
Fried's Criteria			
SGA	84	88	Sheard J.M. et al. E-SPEN Journal 2013;8:e187-e92
SGA	71	99	Sheean P.M. et al. Clin Nutr 2013;32:752–757
NRS-2002	87	44	
NRS-2002 for elderly >70 years	81	96	
In hospital mortality	74	42	Abd-El-Gawad W.M. et al. Clin Nutr 2014;33:1108–1116
Prolonged hospital length of stay	72	37	
Infectious complications	72	31	
Clinical assessment by by two qualified physicians	90	96	Shilpa J. &amp; Kumari K.S. Int J of Adv Res 2014;2:214–221
BMI <20	95	94	
Mid Upper Arm Circumference <22 cm	91	59	
Calf Circumference <31 cm	73	98	
Combined index: malnourished to any degree or at risk of malnutrition according to at least 4 out of 5 of the screening tools (MNA®, MNA® -SF, GNRI, MUST, NRS-2002)	100	61	Baek M.-A. Et al. Nutr Res Pract 2015;9(6):637–643
SGA	93	72	Calleja Fernández A. Et al.
Nutr Hosp 2015;31:2240–2246			
Clinical Status (extensive evaluation(anthropometry, biochemical markers, 3d diet record, CG) by 2 geriatricians)	92	86	Sarikaya D. Et al.
Arch Gerontol Geriatr 2015;61:56–60			
BMI <18.5 kg/m^2^	74	63	Machado R.S.P. etal. BMC Geriatr 2015;15:132
Mid-arm circumference <23 cm	83	77	
Calf circumference <31 cm	67	80	
Body fat (<24% women/<14% men)	59	74	
ICD-10-AM criteria	58	97	Marshall S. Et al. J Acad Nutr Diet 2016;116:785–794
BMI	86	67	Ghimire S P. Et al plos One 2017;12:e0172052
BMI <18.5 kg/m^2^	80	73	Hailemariam H. Et al. BMC Nutrition 2016;2:11
Chang nutritional assessment	63	73	Muñoz Díaz B. Et al. (2019) Family Practice 2019;36:172–178
Serum albumin ≥3.5 g/ml	75	78	Doroudi T. Et al. Int J Prev Med 2019;10:168
BMI <18.5 kg/m^2^	88	90	Woldekidan M.A. et al. South Afr J Clin Nut Online 27 Mar 2020
Mean	80	68	
± SD	13	22	
Range	56 – 100	31 – 99

**Table 16:** MNA® -SF Sensitivity/Specificity against Nutritional assessment parameters &amp; other parameters

**Reference**	**Sensitivity**	**Specificity**	**References**
Detailed nutritional assessment	93	38	Visvanathan R. Et al. Age Ageing 2004;33:260–265
Malnutrition by nutritionist	100	38	Ranhoff AH et al. J Nutr Health Aging 2005;9:221–225.
BMI<23	86	71	
BMI<18.5	100	74.1	Suzana S.J. et al. Malays J Nutr 2007;13:29–44
MUAC (<22cm for women; <23cm for men)	81.8	97.3	
CC (<27.3cm for women; <30.1cm for men)	91	74	
Dietitian assessment	80	90	Harris D.G. et al. J Hum.Nutr Diet 2008;21:3–9
Albumin 3.5 g/dl	44	61	Yamada K. Et al. Am J Clin Nutr 2008;87:106–113
Prealbumin 30 mg/dl	48	67	
BMI < 18.5 kg/m^2^	100	81	
Thigh muscle area/Thigh bone area <10	65	68	
CONUT (controlling nutritional status): albumin, total cholesterol and total lymphocyte count	77	61	Jürschik Jiménez P. Et al. Arch Latinoam Nutr 2009;59(1):38–46
Moderate risk of malnutrition (5–10% unintentional weight loss last 6 months, BMI <20.0 kg/m2) and severe risk of malnutrition (BMI <18.5 kg/m2, weight loss >5% last month or >10% last 6 months)	100	41	Neelemmaat F. Et al. J Clin Nurs2011; 20:2144–2152
BMI &amp; Weight Loss:			Hertroijs D. Et al. J Rehabil Med 2012;44:696–701
1. Severely undernourished: BMI < 20 and/or > 5% unintentional weight loss in the past month and/or > 10% unintentional weight loss in the past 6 months	93	44	
2. Moderately undernourished: BMI 20–22 and/or 5–10% unintentional weight loss in the past 6 months	44	89	
Frailty			
Fried's Criteria	64	79	Dent E. Et al.
J Nutr Health Aging 2012;16:764–767			
SGA as reference	95	78	Sheard J.M. et al.E-SPEN Journal 2013;8:e187-e92
SGA as reference	100	53	Young A.M. et al. Nutrition 2013;29:101–106
Prediction of inadequate energy intake	72	29	
In hospital mortality	73.7	41.5	Abd-El-Gawad W.M. et al. Clin Nutr 2014;33:1108–1116
Prolonged hospital length of stay	72.3	36.7	
Infectious complications	71.6	30.6	
Combined index: malnourished to any degree or at risk of malnutrition according to at least 4 out of 5 of the screening tools (MNA®, MNA® -SF, GNRI, MUST, NRS-2002)	100	49.4	Baek M-H. &amp; Heo Y-R. Nutr Res Pract 2015;9:637–643
Clinical Status (extensive evaluation(anthropometry, biochemical markers, 3d diet record, CG) by 2 geriatricians) Frailty:	94	81	Sarikaya D. Et al. Arch Gerontol Geriatr 2015;61:56–60
Fried's frailty index	94	83.3	Lilamand M. Et al. J Nutr Health Aging 2015:19:570–574
Albumin 3.5 g/dl	68	61	Zhou J. Et al. Nutr J 2015;14:68
ICD-10-AM criteria	100	23	Marshall S. Et al. J Acad Nutr Diet 2016;116:795–801
Muscle wasting: i. E., appendicular skeletal muscle mass two standard deviations (SD) below the mean of a healthy young reference group aged 18–40 years	74	54	Saitoh M. Et al. Wien Klin Wochenschr 2016;128:497–504
ESPEN diagnostic criteria	100	63	Van der Sijp M.P.L. et al. Injury 2018;49:2239–2243
ESPEN diagnostic criteria	58	65	Sánchez-Rodríguez D, et al. Arch Gerontol Geriatr 2018;76:210–214
SENPE-SEDOM coding criteria	96	52	Castro-Vega I. Et al. Nutr Hosp 2018;35(2):351–358
Clinical dietitian's assessment of malnutrition	53	68	Thomas J. Et al. Br J Nutr 2019;122:689–697
SGA as reference	79	88	Joaquin C. Et al. Clin Nutr. 2019;38:2740–2746
Serum albumin ≥3.5 g/ml	63	65	Doroudi T. Et al. Int J Prev Med 2019;10:168
BMI <18.5 kg/m^2^	86	90	Woldekidan M.A. et al. South Afr J Clin Nut Online 27 Mar 2020
Research nutritionist risk rating score	95	89	Pavlović J.R. et al. (2020) Public Health Nutr on line September 01, 2020
Mean	81	63	
± SD	18	20	
Range	44–100	23–90

**Table 17:** MNA® -SF Sensitivity/Specificity against MNA®

**Reference**		**Sensitivity**	**Specificity**	**References**
MNA®		96	98	Rubenstein LZ et al. 2001
MNA®		86	89	Cohendy R et al. 2001
MNA®	Community	74	95	Borowiak E. &amp; Kostka T. 2003
	Insitution	64	100	
MNA®		86	94	Kuzuya M et al. 2005
MNA®		100	95	Charlton K.E: et al. 2007
MNA®		89	82	Wikby et al. 2008
MNA®		85	89	Cuervo M. Et al Public Health Nutr 2009;12:82–90
MNA®		89	44	Charlton K.E. et al. J Nutr Health Aging 2010;4:622–628
MNA®		95	64	Calvo I. Et al. Nutr Hosp 2012;27:1619–1625
MNA®		72	98	Sheean P.M. et al. Clin Nutr 2013;32:752–757
MNA®		81	97	MNA® -SF CC Donini L.M. et al. J Nutr Health Aging 2013;17:332–328
MNA®		96	79	Young A.M. et al. Nutrition 2013;29:101–106
MNA®	Urban Community	100	97	MNA® -SF BMI Kostka J. Et al. J
	Rural Community	92	94	Nutr Health Aging 2014;18:366–371 MNA® -SF CC
	Insitution	83	96	
MNA®	Urban Community	88	97	
	Rural Community	81	90	
	Insitution	74,1	94	
MNA®		94	83	Lilamand M. Et al. J Nutr Health Aging 2015:19:570–574
MNA®		96	56	Donini L.M. et al. J Am Med Dir Assoc 2016;17(10):959.e11-959.e18
MNA®		73	87	Montejano Lozoya R. Et al. Peerj 2017;5:e3345
MNA®		90	78	MNA® -SF BMI Dent E. Et al. Australas J Ageing 2017;36:E8-E31
		95	65	MNA® -SF CC
MNA®		71	94	Joaquín C. Et al. Clin Nutr. 2019;38:2740–2746
MNA®		95	63	Holvoet E. Et al. Plos ONE 2020;15(3):e0229722
Mean		87	85	
± SD		10	15	
Range		64–100	44 – 90	

The MNA® appears also to be useful as primary criteria for intervention studies, and move positively in several major studies (56, 89, 98, 330, 331, 332, 333, 334).

Further, the MNA® appears useful to measure frail older persons, especially when the MNA® is between 17 and 23.5 (27, 286, 303, 335, 336). Malnutrition and physical frailty seem to be strongly related, however, they should be assessed separately within the geriatric assessment (313, 317, 326, 337, 338, 339, 340, 341).

### MNA® use in clinical practice

At least 22 Expert groups included the MNA® in new clinical practice guidelines, national or international registries (113, 116, 131, 132, 136, 151, 189, 190, 319, 324, 342, 343, 344, 345, 346, 347, 348, 349, 350, 351, 352, 353, 354, 355, 356, 357, 358, 359, 360, 361, 362, 363, 364, 365, 366, 367, 368, 369, 370, 371, 372, 373, 374, 375, 376, 377). New Global Leadership in Malnutrition (132) consensus publication outlines the diagnostic criteria for malnutrition, for application in clinical settings (131, 151). GLIM Committee involved major global clinical nutrition societies: ASPEN, ESPEN, FELANPE, PENSA representatives (Tables 18). 42 Electronic Health Record Software Companies have incorporated MNA® in software, 22 APPS for Smartphones, tablets have incorporated MNA® (Table 19).

**Table 18:** MNA®: Practicalities

At least 22 Expert groups included the MNA® in new Clinical Practice Guidelines, National or International registries, such as:	
•	World Health Organization Integrated Care for Older People (ICOPE) guidelines.
•	Swedish National Board of Health and Welfare document about Malnutrition
•	Statement on Nutrition Assessment and Management of Heart Failure Patients in Japan
•	Use in protocol of patients with Steinert's dystrophy (Spain)
•	Oral Management by Dental Treatment and Nutritional Management for the Support of Oral Feeding in Elderly People with Dementia (Japan)
•	iCertus Health, best practice guidelines for wound care
•	American Dietetic Association's Evidence Analysis Library toolkit for Unintended Weight Loss for Older Adults
•	Associazione Italiana di Dietetica e Nutrizione Clinica (ADI), Associazione Medici Diabetologi (AMD), and Società Italiana de Diabetologia (SID). Use in Recommendations 2013–2014-Medical Nutrition Therapy in Diabetes Mellitus. (Italy)
•	Austrian Society of Geriatrics and Gerontology (OGGG) Geriatric Assessment Guide Quebec Health Ministry's nutrition card
•	Canadian Patient Safety Institute program, Safer Healthcare Now (http://www.patientsafetyinstitute.ca) (CA)
•	Central Coast Local Health District care pathway «older adult weight and nutrition“
•	National Meal Guidelines resource for Australian Meals on Wheels Association
•	Nutrition UP 65 Project for older Portuguese population

**Table 19:** MNA®: Incorporation of the MNA® into electronic and applications software

42 Electronic Health Record Software Companies have incorporated MNA® into the software, and 22 Apps for smartphones and tablets have incorporated it as well, such as:	
• Evolve Health Cloud (USA) electronic platform that includes medicare annual wellness visit	• Health Communication Online tool (Dutch)
• 360medical software medical library for physicians (France)	• Health Pathways, online health information portal for local GPs (Australia)
• Android app of screening scales available on SSPEN website	• iCertus Health — a distillation of best practice guidelines for wound care
• California Association of Healthcare Facilities (CAHF) online toolkit for care transitions	• Linkcare, open platform used in observational trials (Spain)
• Care partners assessment tool package at bedside wound care (Canada)	• MaNeEL consortium (Europe) 22 research groups in 7 countries (Europe)
• CHECKWARE software instruments page (Norway)	• Nutritional Medicine Pocket app by Brett and Mechanick (Germany)
• Endo Education Co. smartphone app — iswallow 2 app (iS2 platform) (US)	• Optum website includes interactive Self-MNA® (US)
• Euromedice Ediciones Medicas, S.L. (geriatric assessment tools app for geriatricians)	• Regional Geriatric Program of Toronto — Use in toolkit on nutrition for healthcare professionals and older adults
• GAVON, commercial web-based functional performance assessment solution (Finland)	• Scorso by DIM3 malnutrition app for hospitals and nursing homes (Belgium)
• GCS D-SISIF software (France)	• Smartphone app for French SPEN (France)
• GrandCARE electronic caregiving tool	• vSim for Nursing / Gerontology on-line tool (US)

Notably, the MNA® screening tool evaluates items that are similar to relevant criteria that GLIM established for the diagnosis of malnutrition (202, 378). Furthermore for the evaluation of malnutrition in the elderly, the MNA® with its two step procedure a) screening with MNA®-SF and b) nutritional status evaluation with the MNA® full form can be completed with evaluation of nutritional makers (e.g. as serum C-reactive protein and transthyretin (prealbumin) (379, 380, 381, 382, 383, 384, 385, 386)) or the GLIM criteria (131, 206, 207, 223, 376, 387). This is of importance to separate malnutrition from inflammation, when undernutrition is due to disease related cachexia (174, 380, 383, 388, 389, 390, 391). MNA® fulfill the two steps, screening followed by assessment, required by the GLIM criteria procedure for the diagnosis of protein-energy malnutrition in elderly (376, 377), and provides guidance for nutritional intervention. Elderly with malnutrition or at risk of malnutrition should have a nutritional intervention with multidisciplinary team in order to support adequate dietary intake, maintain or increase body weight and/or improve functional and clinical outcome (184, 189, 190, 363, 392).

### MNA® and Healthy aging

**Figure 3:**
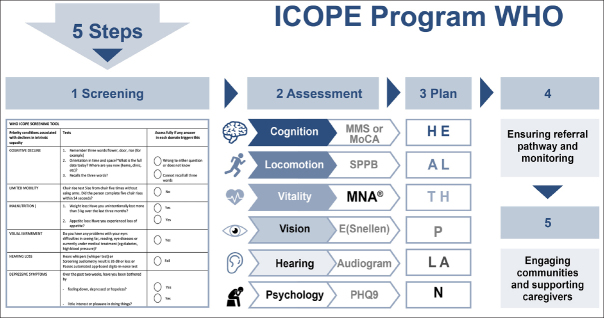
W.H.O Icope Pogram

For the W.H.O Healthy aging is the capacity to maintain function, to be able to do what we value. The ICOPE step 1 includes some nutrition assessment (weight loss, poor appetite), and MNA® is part of the ICOPE program step 2, Integrated care of older persons (figure 3) to maintain functions in older adults (393, 394, 395). Our practice needs really to move to prevention and ambulatory care. We need to provide good nutrition to the senior citizen and monitor their nutritional status, the aims of ICOPE Monitor apps part of the Inspire program is to monitor the main function including nutrition in older adults. The links between Nutrition and Geroscience for a healthy aging have to be studied (396, 397).

Perspective for the future have of course to treat undernutrition in sick old adults; however, the urgent need is to target the frail older adults more likely to have weight loss and poor appetite. To do it, program for early detection of the risk of malnutrition should be implemented as it is in development in Netherlands (80, 81, 115, 184), the NUDAD (Nutrition, the unrecognized determinant in Alzheimer's disease) study (139, 398) and within the Integrated Care for Older People (ICOPE) with the implementation of the INSPIRE study (213, 394, 399). Further the geriatric assessment to be comprehensive should include the MNA®-SF as nutrition parameters and all the elderly detected at risk of malnutrition or malnourished should be further evaluated with GLIM criteria, and MNA® to be able to implement a nutritional intervention. The best MNA® score to predict healthy aging is still to be determined
